# Comparative genomic and transcriptomic analyses of the Fuzhuan brick tea-fermentation fungus *Aspergillus cristatus*

**DOI:** 10.1186/s12864-016-2637-y

**Published:** 2016-06-07

**Authors:** Yongyi Ge, Yuchen Wang, YongXiang Liu, Yumei Tan, Xiuxiu Ren, Xinyu Zhang, Kevin D. Hyde, Yongfeng Liu, Zuoyi Liu

**Affiliations:** College of Agriculture, Guizhou University, Guiyang, Guizhou 550025 China; Guizhou Key Laboratory of Agricultural Biotechnology, Guiyang, Guizhou 550006 China; College of Life and Science, Guizhou University, Guiyang, Guizhou 550025 China; Guizhou Institute of Biotechnology, Guiyang, Guizhou 550006 China; Ecological Engineering College, Guizhou University of Engineering Science, Bijie, Guizhou 551700 China; Institute of Microbiology, State Key Laboratory of Mycology, Chinese Academy of Sciences, Beijing, 100101 China; Institute of Excellence in Fungal Research, Mae Fah Luang University, Chiang Rai, 57100 Thailand; Big Genome Institute, Shenzhen, 518083 China; Guizhou Academy of Agricultural Sciences, Guiyang, Guizhou 550006 China

**Keywords:** *Aspergillus cristatus*, Genome, Mating-typing gene loci, HOG pathway, Mycotoxin

## Abstract

**Background:**

*Aspergillus cristatus* is the dominant fungus involved in the fermentation of Chinese Fuzhuan brick tea. *Aspergillus cristatus* is a homothallic fungus that undergoes a sexual stage without asexual conidiation when cultured in hypotonic medium. The asexual stage is induced by a high salt concentration, which completely inhibits sexual development. The taxon is therefore appropriate for investigating the mechanisms of asexual and sexual reproduction in fungi. In this study, *de novo* genome sequencing and analysis of transcriptomes during culture under high- and low-osmolarity conditions were performed. These analyses facilitated investigation of the evolution of mating-type genes, which determine the mode of sexual reproduction, in *A. cristatus*, the response of the high-osmolarity glycerol (HOG) pathway to osmotic stimulation, and the detection of mycotoxins and evaluation of the relationship with the location of the encoding genes.

**Results:**

The *A. cristatus* genome comprised 27.9 Mb and included 68 scaffolds, from which 10,136 protein-coding gene models were predicted. A phylogenetic analysis suggested a considerable phylogenetic distance between *A. cristatus* and *A. nidulans*. Comparison of the mating-type gene loci among *Aspergillus* species indicated that the mode in *A. cristatus* differs from those in other *Aspergillus* species. The components of the HOG pathway were conserved in the genome of *A. cristatus*. Differential gene expression analysis in *A. cristatus* using RNA-Seq demonstrated that the expression of most genes in the HOG pathway was unaffected by osmotic pressure. No gene clusters associated with the production of carcinogens were detected.

**Conclusions:**

A model of the mating-type locus in *A. cristatus* is reported for the first time. *Aspergillus cristatus* has evolved various mechanisms to cope with high osmotic stress. As a fungus associated with Fuzhuan tea, it is considered to be safe under low- and high-osmolarity conditions.

**Electronic supplementary material:**

The online version of this article (doi:10.1186/s12864-016-2637-y) contains supplementary material, which is available to authorized users.

## Background

Chinese commercial tea is classified as green, oolong, black, white, yellow and dark teas, according to the manufacturing process used. Fuzhuan brick tea is a type of dark tea that has been produced for more than 400 years [1]. The production of Fuzhuan brick tea exceeded 10 billion yuan in 2014, and it is very popular in China and north-eastern Asia.

**Fig. 1:**
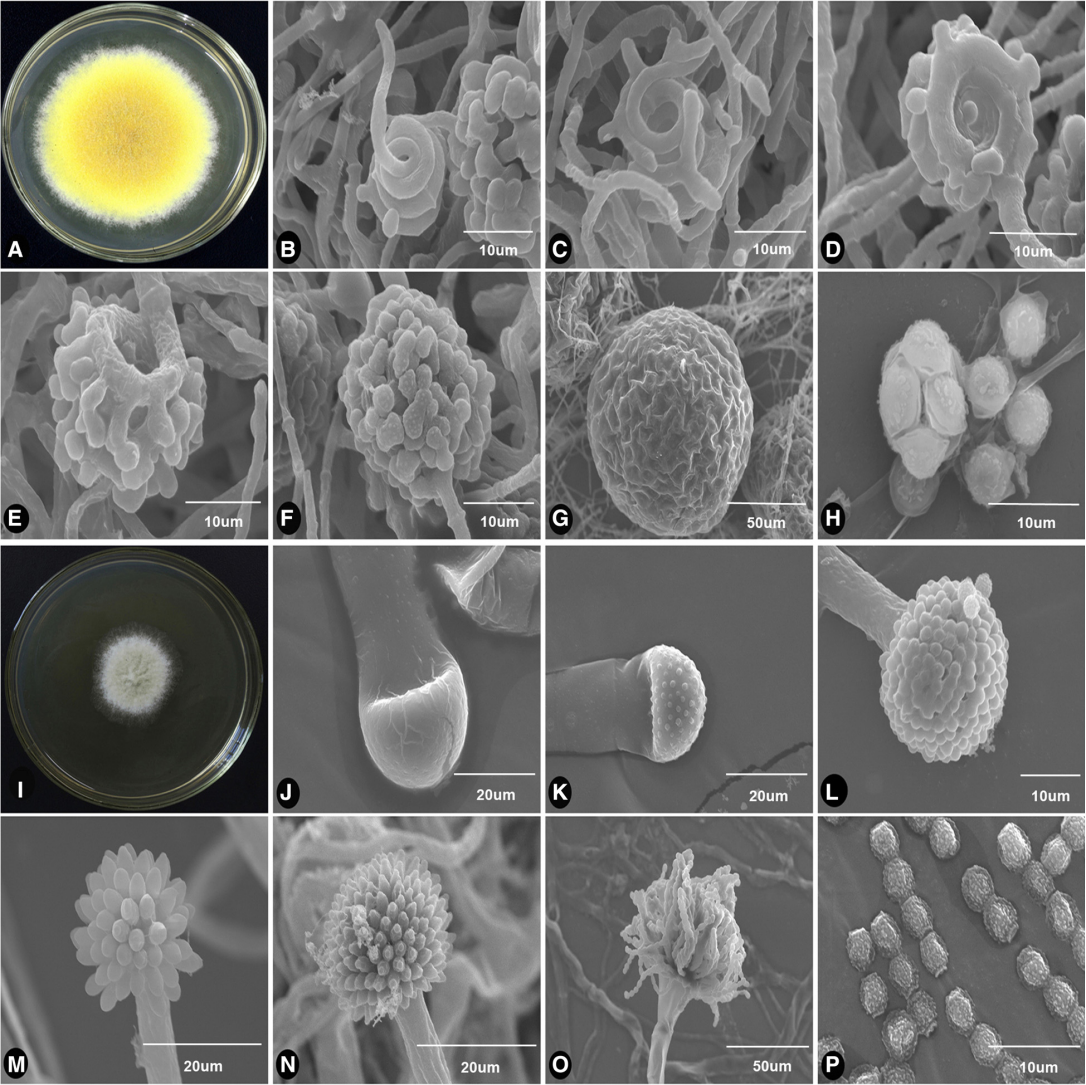
Sexual and asexual morphs of *Aspergillus cristatus*. **a**, **i** Dissecting microscopy; **b**-**h**, **j**-**p** Scanning electron microscopy. Sexual morph: colony phenotype in 0.5 M NaCl (**a**), ascogonium during the early and late stages (**b**-**e**), cleistothecium (**f**-**g**), ascus and ascospore (**h**). Asexual morph: colony phenotype in 3 M NaCl (**i**), conidiophores during the early and late stages (**j**-**o**), conidia (**p**)

Fuzhuan brick tea involves microbial fermentation, which exerts a major effect on its organoleptic qualities and health properties [2, 3]. Various fungal taxa are important during the production of Fuzhuan brick tea, which is produced under controlled temperature and moisture conditions [4]. *Aspergillus*, *Eurotium* and *Penicillium* species are the main fungal taxa isolated during fermentation. *Aspergillus cristatus* is the dominant taxon, termed the “Golden Flower Fungus” because of its yellow cleistothecium colour (Fig. 1) [5].

Aspergilli have served as model organisms in genetic studies because of their multifaceted life cycle [6]. The majority of *Aspergillus* species (approximately two-thirds) reproduce only asexually, whilst those that exhibit sexual cycles are overwhelmingly homothallic in nature, there are few heterothallic species [7, 8]. Aspergilli are considered good candidates for genetic studies of reproduction in fungi [6, 9]. In the classical era of genetics, the optimal method of identifying gene function was screening for mutants that exhibit defective phenotypes related to their function. However, isolation of sexual reproduction mutants was problematic because of the priority production of conidia [10]. A range of genes involved in sexual reproduction have been identified in several *Aspergillus* species [9].

*Aspergillus* can develop asexual or sexual spores depending on growth conditions. High concentrations of salts, such as sodium chloride or potassium chloride, induce asexual reproduction but inhibit sexual spore formation in Aspergilli [11, 12]. Osmotic pressure also plays a key role in *A. cristatus* sporogenesis [13]. *Aspergillus cristatus* reproduces sexually only when grown in tea bricks and during culture in hypo-osmolar medium (Fig. 1a–h). Increased osmotic pressure results in greater numbers of asexual spores and fewer sexual spores. In ≥3 M NaCl conditions, only asexual conidia are produced (Fig. 1 i–p), and sexual reproduction is completely inhibited. *Aspergillus cristatus* employs different sexual and asexual reproductive strategies under different salt stress conditions, providing a robust genetic system for the study of eukaryotic sex development and cell biology.

Fuzhuan brick-tea is produced only in China [14]. As it is popular with certain ethnic groups in China and northeastern Asia, it is important to establish that *A. cristatus* does not produce carcinogenic mycotoxins, because this fungus dominates the fermentation process during brick-tea production. Many *Aspergillus* species produce mycotoxins by means of the mycotoxin pathway gene cluster, which comprises several genes [15–20]. Most of the clusters contain one or several central biosynthesis genes encoding extremely large, multidomain, multimodular enzymes belonging to the polyketide synthases (PKSs) or non-ribosomal peptide synthetases (NRPSs) [21]. Consequently, in this study, we focused on analysing the relationships between mycotoxin gene clusters and end products.

In this paper, we investigated the relationship between the HOG pathway and osmotic pressure, verified the safety of *A. cristatus* under low- and high-osmotic pressure conditions, and established the evolutionary patterns of the mating-type genes through genome sequencing and RNA-Seq transcriptomic data from *A. cristatus* at two developmental stages. This is the first high-quality genome sequence of *A. cristatus* to be published and the first report of its safety using genomic data mining. This study may serve as a model for further investigations of the relationship between osmotic pressure and reproduction mode.

## Results and discussion

### Genome sequencing and assembly

**Fig. 2:**
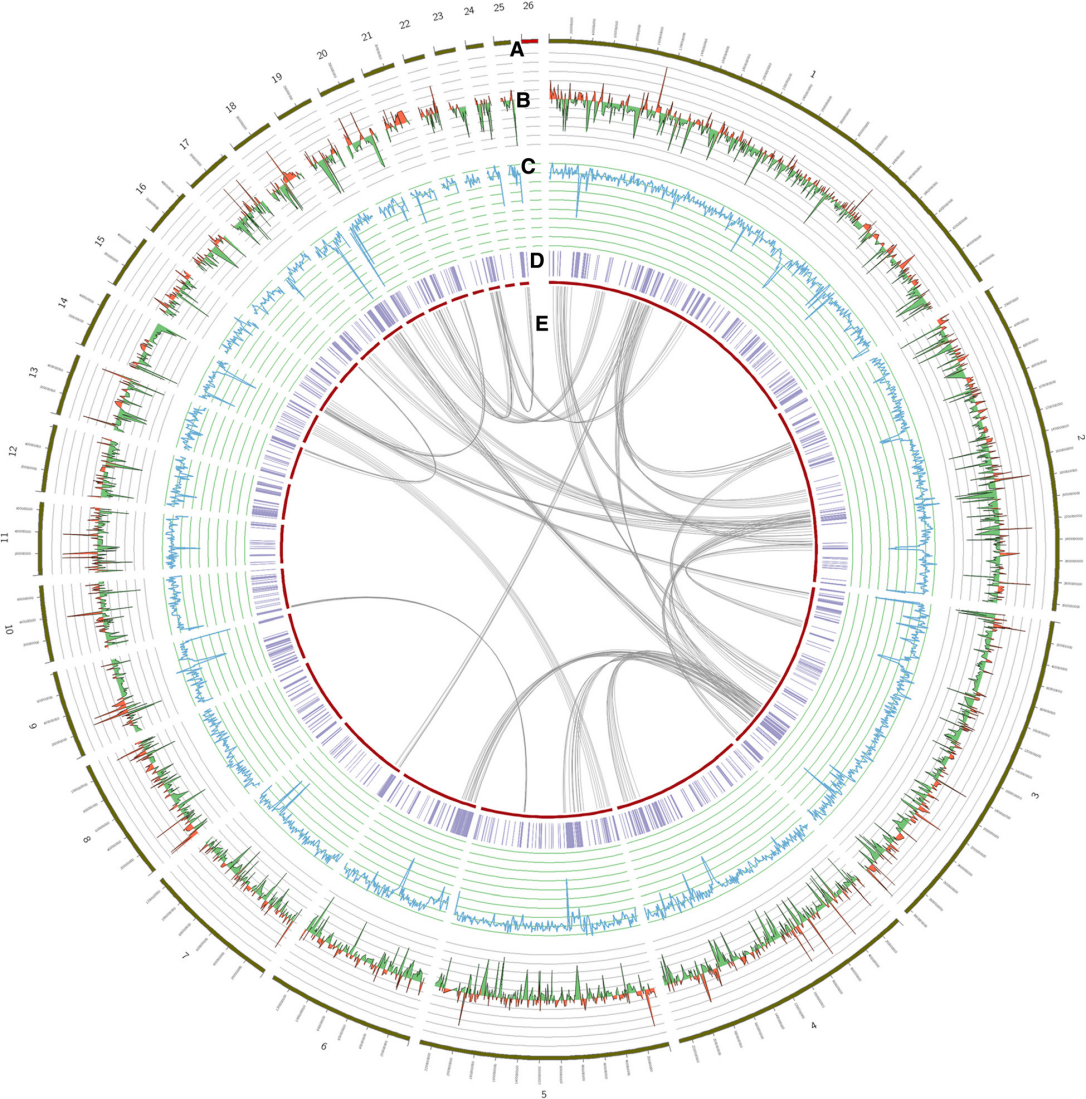
Circular representation of the *Aspergillus cristatus* genome. The following data are shown (from the outside, in): **a** Size of scaffolds > 15 kb. **b** Differential expression as the log2 ratio of expression at high salinity (3 M NaCl), with increased expression in red and decreased expression in green. **c** GC content of each scaffold. **d** Locations of transposable elements. **e** Gene duplications and links. Linked locations were determined by aligning the predicted proteins to the genome using Exonerate (cut-off: 1e-10) [79]

The genome of *A. cristatus* was sequenced using a whole-genome shotgun approach. A total of 3,489 Mb raw sequence data were generated from the Illumina Hiseq 2000 platform at BGI-ShenZhen. After filtering, the total assembly size of the genome of *A. cristatus* was 27.9 Mb, which was assembled into 168 contigs and 68 scaffolds, with an N50 length of 2.3 Mb (Fig. 2, Table 1). With the exception of *A. clavatus*, *A. rambellii* and *A. ruber,* the genome of *A. cristatus* is smaller than other sequenced *Aspergillus* genomes [22] (Additional file 1: Table S1).

**Table 1:** General feature of *Aspergillus cristatus* genome assembly

Genome	Value
Nuclear genome	
General information	
Size (Mb)	27.9
Number of scaffolds	68
N50 (bp)	2308221
G + C content (%)	49.68
Coding (%)	51.92
Protein coding genes	10136
Mean gene length (bp)	1573.31
Genes with intron (%)	9.15
Exons	
Total exon number	30219
Total exon length	14486784
Mean number per gene	2.98
Mean length (bp)	479.39
G + C content (%)	53.72
Introns	
Total intron number	20083
Total intron length (bp)	1460267
Mean number per gene	1.98
Mean length (bp)	72.71
G + C content (%)	44.54

### Gene prediction and annotation

A total of 10,136 genes were predicted via *ab initio* and homology-based analyses. The gene density was 2.98 kb per gene, which is higher than those of other sequenced *Aspergillus* species (Additional file 1: Table S1). In *A. cristatus*, the annotated coding regions accounted for 51.92 % of the genome, with an average coding length of 1,573 bp and 2.98 exons per gene; the average exon length was 479 bp. The overall GC content was 49.68 %, while the average GC content of the open reading frames was 53.72 % (Table 1).

Gene ontology analysis categorised the gene set into 443 functional groups. Subsets of these functional groups were annotated within the “mating projection” category. Kyoto Encyclopaedia of Genes and Genomes (KEGG) analysis was used to assign 5,159 genes to 311 pathways [23]. Thirteen genes were predicted to be involved in the HOG signalling pathway, with the exception of *Sln1*, which controls adaptation to different osmolarities [24] (Table 2).

**Table 2:** HOG MAP-kinase pathway genes in *Aspergillus cristatus* and the expression under different osmolarities

*S. cerevisiae*	*A. nidulans*	*A. cristatus*	Conidia/Asexual (log2 FPKM)	Ascospore/Sexual (log2 FPKM)	*P*-value
Ctt1	catA	SI65_01337	−1.302475	−2.95113888	1.03E-07
Msn2	AN4013	SI65_01521	3.5055734	3.439995398	5.86E-26
ssk2	sskB	SI65_02844	2.8020422	2.834553469	1.26E-31
Msn4	brlA	SI65_02778	3.9057937	6.439433647	6.09E-32
Ste11	steC	SI65_03137	2.8353663	5.874860888	9.51E-185
ssk1	sskA	SI65_09147	4.1617507	4.469430179	0.000218894
Pbs2	PbsB	SI65_06242	5.001496	5.74608968	1.16E-27
Sho1	shoA	SI65_09148	8.595541	8.395251352	6.76E-32
Mcm1	mcmA	SI65_10214	5.9391184	7.177598929	0.01221206
Ste20	AN2067	SI65_07597	5.1717752	6.7099798	0.000886426
ypd1	YpdA	SI65_00763	6.5681294	5.056717908	0
Glo1	AN4174	SI65_07143	6.7967535	6.2913457	7.64E-12
Hog1	HogA	SI65_07698	7.2766828	7.036393435	1.36E-63

### Phylogenetic relationships

**Fig. 3:**
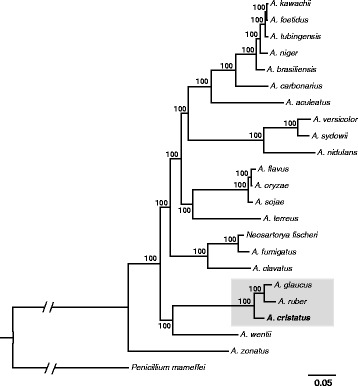
Phylogenetic relationships among *Aspergillus genomes*. The grey rectangle represents the clade in which *Aspergillus cristatus* is located. A total of 1,034 single copy orthologous proteins were concatenated and a phylogenetic tree constructed using the RaxML 7.2.8 software [69], with the best model, which was simulated with ProtTest 1.4 [70], 1000 bootstrap replicates were used

The phylogenetic relationships between filamentous fungi have been established based on ribosomal DNA sequences or single-gene families [25]. We selected a set of 1,034 single-copy predicted orthologous genes from *A. cristatus* and 22 sequenced genes from filamentous fungi. These orthologous genes were used to construct a maximum-likelihood tree (Additional file 1: Table S2). *Penicillium marneffei* was used as an outgroup taxon to root the tree. The topology (all internal branches had 100 % bootstrap support) of this tree was consistent with previous reports [22]. *Aspergillus cristatus*, *A. glaucus* and *A. ruber* clustered within the same clade, indicating closer genetic relationships than that with *A. nidulans* (Fig. 3). The teleomorph of three species are *Eurotium* spp., and they are homothallic. These microorganisms are widely distributed in the natural environment, can adapt to high concentrations of sodium chloride and other ions, and thus are a valuable resource for investigation of stress resistance mechanisms [26, 27].

### Sex and evolution of mating-type loci

Sexual reproduction in ascomycetes is governed by two mating-type genes: one gene encodes a protein with an alpha-box domain (mating-type gene *MAT1-1-1*), and the other encodes a protein with a high mobility group (HMG) domain (mating-type gene *MAT1-2-1*) [28]. The sexual development of the genus *Aspergillus* is mainly homothallic, and the two mating-type genes are located on different chromosomes [6]. Heterothallic fungi have only one mating-type gene and require a partner with a different mating-type gene. The two mating-type genes of heterothallic fungi typically occupy the same chromosomal location in different haploid genomes but are not obviously related in terms of structure or common descent; these genes are termed idiomorphs [29].

Two models for the evolution of the *MAT* locus in Aspergilli have been proposed (Fig. 5a, b). The first model is evolution of heterothallism from homothallism [30]. The second model is evolution of homothallism from heterothallism [31]. In the first model, the shared homothallic ancestor included two adjacent genes (*MAT1-1-1* and *MAT1-2-1*), which are flanked by the *SLA2* and *APN1* genes. The alpha box and HMG domain genes in one lineage were located on different chromosomes flanked by either the *SLA2* or *APN1* gene through chromosomal breakage, resulting in heterothallic species. In addition, the ancestor evolved into heterothallic species with an alpha box or HMG domain gene at the same locus due to chromosomal segregation and gene loss. In the second model, the shared heterothallic ancestor contained either the alpha box or the HMG domain gene at the same locus, flanked by the *SLA2* and *APN1* genes. The alpha box and HMG genes of the ancestor separated to different chromosomes flanked by the *SLA2* and *APN1* genes, respectively, due to chromosomal breakage, translocation and rearrangement, resulting in the evolution of homothallic species. Moreover, when the ancestor underwent gene duplication and chromosomal translocation, it evolved into other homothallic species (*Neosartorya fischeri*), which contained the alpha box domain gene at the original loci flanked by *SLA2* and *APN1*, while the HMG gene was located at a separate locus flanked by d*SLA2* and d*APN1*, which are pseudogenes of *SLA2* and *APN1*, respectively [31]. The sequence upstream of the *N. fischeri* MAT2 locus contains numerous regions with sequence similarity to transposase genes from other fungi.

The annotation results of the *A. cristatus* genome showed that *MAT1-1-1* (*SI65_05562*) and *MAT1-2-1* (*SI65_06277*) are distributed in scaffolds 5 and 6, respectively. The BLASTp analysis indicated that these genes are present as a single copy in the *A. cristatus* genome. Fgenesh + analysis suggested that *MAT1-1-1* contains two exons and an alpha-box sequence and encodes 384 amino acid residues, while *MAT1-2-1* contains three exons and one HMG-box sequence and encodes 357 amino acid residues. The conserved amino acid sequences of the two genes were subject to a BLAST search against those of other *Aspergillus* species using Jalview version 2.0 [32] (Additional file 2: Figure S1). The results demonstrated that *MAT* genes are conserved in *A. cristatus*.

**Fig. 4:**
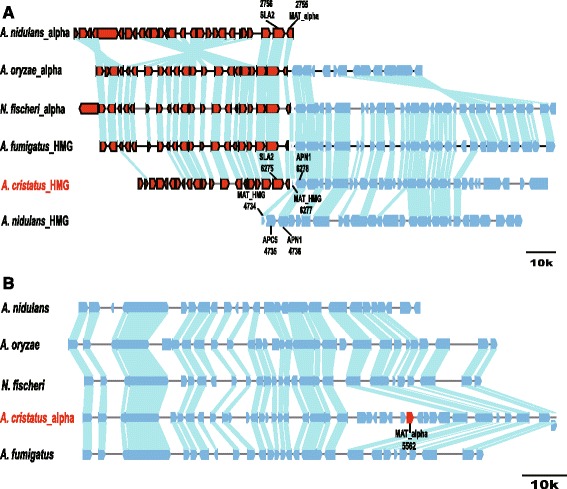
Comparison of *Aspergillus MAT* loci. Coding regions are indicated by arrows, with the direction of the arrow denoting the direction of transcription. **a** The *Aspergillus cristatus MAT*-HMG locus is co-linear with other *Aspergillus MAT* loci. Red genes show orthologs from the left flank (as drawn) of the *A. nidulans* alpha locus with the left flanks of *A. oryzae*, *N. fischeri* and *A. cristatus* loci. Blue genes indicate orthologs to the right flank of the *A. nidulans* HMG locus. Genes labelled and outlined in black are associated with *MAT* loci in other fungi. **b** The *A. cristatus MAT-*alpha locus has no collinearity with any other *Aspergillus* species. The red gene indicates the *MAT*-alpha gene of *A. cristatus*

**Fig. 5:**
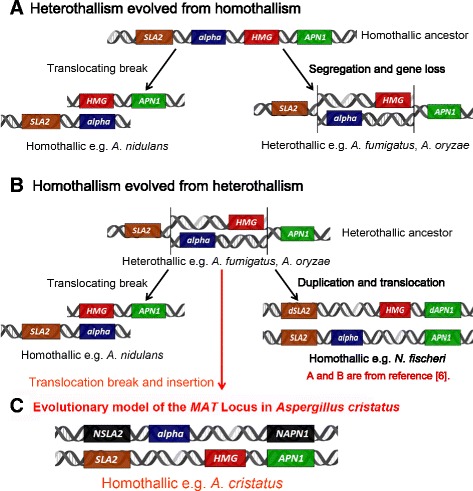
Evolutionary model of the *MAT* locus in *Aspergillus cristatus*. **a** The mode of evolution of *MAT* loci from homothallic to heterothallic species within the genus *Aspergillus*; **b** The mode of evolution of *MAT* loci from heterothallic to homothallic species within the genus *Aspergillus*; **c** evolutionary model of *MAT* loci in *A. cristatus*, the *HMG* (MAT2) locus is flanked by *SLA2* and *APN1*, and the *alpha* (MAT1) locus is flanked by *NSLA2* and *NAPN1*, *NAPN1* and *NSLA2* indicated that the protein flanking the MAT1 locus has no homology with *SLA2* and *APN1*. **a** and **b** are from reference [6]. **c** Model of the evolution of *MAT* loci in *A. cristatus*

BLAST analysis of the flanking regions of *A. cristatus MAT 1-2-1* revealed the presence of putative cytoskeleton assembly control (*SLA2*) and DNA lyase (*APN1*) genes upstream and downstream of *MAT 1-2-1*, respectively. The upstream and downstream regions of the *A. cristatus* MAT 2 locus exhibit collinearity with the upstream and downstream regions of the MAT 1 locus of *N. fischeri* (Fig. 4a). However, analysis of the flanking genes of the *A. cristatus* MAT1 locus showed that these genes have no homology to *SLA2* and *APN1* (Fig. 4b). The sequences flanking the *A. cristatus* MAT1 locus encode proteins that have no homology with *SLA2* and *APN1*; therefore, these loci were termed *NSLA2* and *NAPN1* to distinguish them from *SLA2* and *APN1* (Fig. 5c). In addition, the sequences upstream and downstream of the *A. cristatus* MAT1 locus did not encode transposase genes with similarity to those of other fungi. These results indicated that the evolutionary model of the MAT loci of *A. cristatus* is different from those of other *Aspergillus* species, possibly due to gene translocation breakage and insertion (Fig. 5c). This model supports the evolution of homothallism from heterothallism.

### MAPK signalling transduction

The cell surface and nucleus of eukaryotic cells receive and respond to environmental signals via the MAPK pathway, which is a highly conserved eukaryotic signal transduction system [33]. This pathway orchestrates cell growth, morphogenesis and cell division in response to hormones, stress and other abiotic signals [25]. In the model yeast *Saccharomyces cerevisiae*, four signalling pathways are associated with the MAPK pathway: the pheromone pathway, the hypotonic pathway, the high-osmolarity pathway and the starvation pathway. Among these, the HOG pathway is involved in responses to osmotic pressure, and its activation depends on the induction of several genes in response to increased osmotic pressure [34].

In *S. cerevisiae*, the HOG pathway includes two branches: the Sln1-branch and the Sho1-branch. Regulation of these branches converges on the MAPK kinase (MAPKK) Pbs2 [35]. Under normal conditions, Sln1, a sensor histidine kinase, is constitutively activated by autophosphorylation and subsequently phosphorylates the phosphotransfer protein Ypd1, which, in turn, transfers phosphate to the Ssk1 response regulator. Ssk1 is phosphorylated and inactive under low-osmolarity conditions, which blocks activation of the Pbs2 MAPKK-Hog1 MAPK system. In response to stress, the two-component phosphorelay system is rapidly repressed, resulting in the activation of Ssk2 and Ssk22, which activate Pbs2 and Hog1 [36]. Under high-osmolarity conditions, Sho1, which contains four transmembrane domains and a carboxy-terminal SH3 domain, utilises Ste20 and Ste50 to activate the MAPKK kinase Ste11, which then activates Pbs2 [37, 38]. Pbs2 then phosphorylates the MAPK Hog1, resulting in the translocation of Hog1 into the nucleus and induction of the transcription of a large number of genes, some of which are responsible for glycerol production [39].

As in *S. cerevisiae*, the HogA (SakA) pathway of *A. nidulans* is activated in an osmotic and oxidative manner [40, 41]. Interestingly, a *sakA*-null mutant showed only slight sensitivity to high osmolarity stress, and PbsB (a homolog to *S. cerevisiae* Pbs2) in *A. nidulans* lacks the Pro-rich motif necessary for binding to Sho1p. This indicates that osmo-regulation in *A. nidulans* differs from that in yeast [42].

**Fig. 6:**
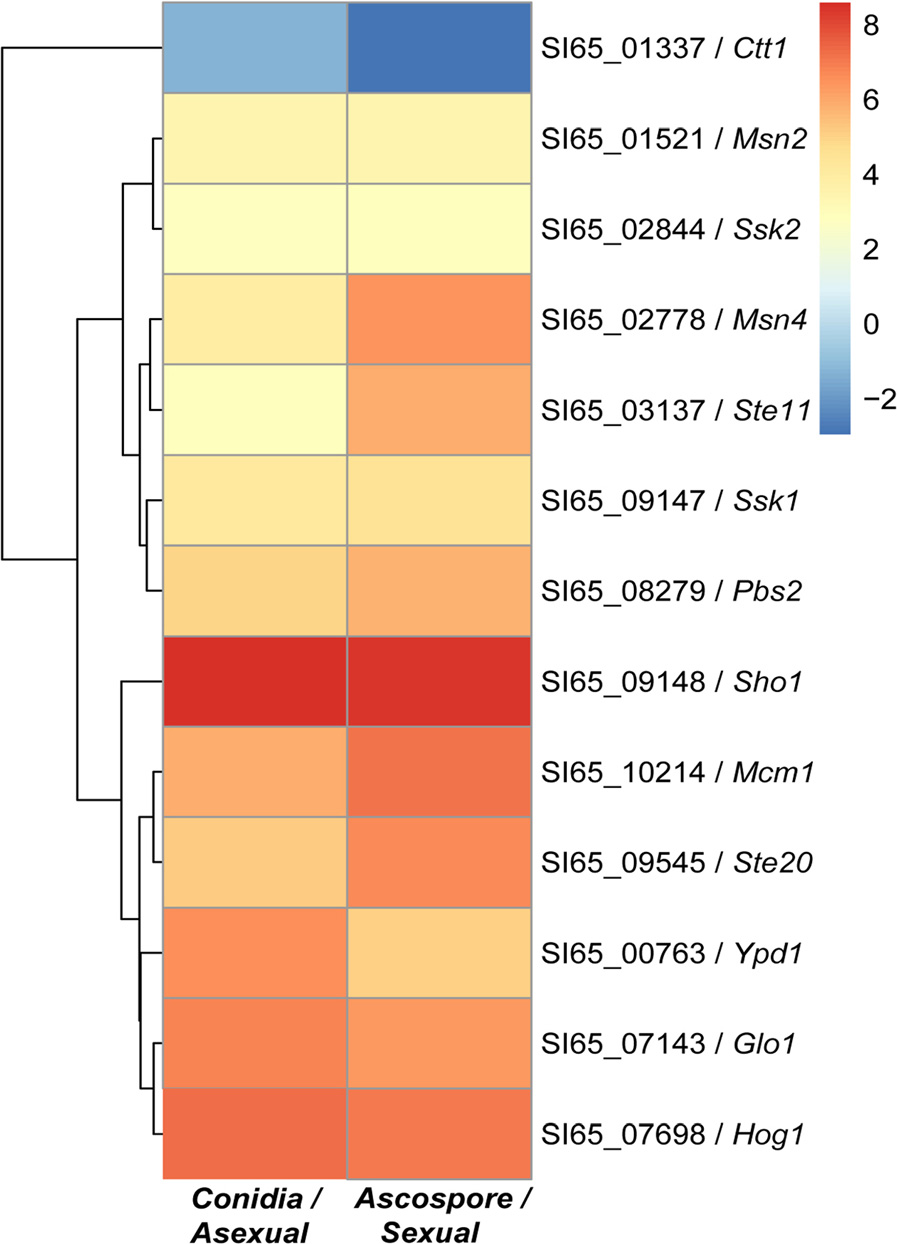
Expression of HOG MAPK pathway genes according to osmolarity. Thirteen genes of the HOG pathway are shown, and background colour changes from blue to red indicate changes in log2 FPKM values (−2, 0, 2, 4, 6, 8; upper right) of gene expression levels under low and high osmolarity conditions. Numbers at right, such as SI65_01337/Ctt1, are Gene IDs or names. Treatment conditions are detailed beneath the images

*A. cristatus* can grow and develop under both low- and high-osmolarity conditions; moreover, most HOG pathway genes in *S. cerevisiae* have homologs in the *A. cristatus* genome, with the exception of *Sln1*. Therefore, we hypothesised that the expression of components of the HOG MAPK cascade pathway would respond to a change in osmotic pressure. To test this hypothesis, we used RNA-Seq to compare gene expression levels in *A. cristatus* cultured in 0.5 M NaCl (sexual stage developed) and 3 M NaCl (asexual stage developed). Interestingly, there was no significant change in the level of expression of most key genes in the HOG pathway—such as *sho1*, *hog1*, and *ste20*—between high and low osmotic pressure conditions (Fig. 6), indicating that the HOG MAPK cascade pathway is not involved in the response to changes in the osmotic pressure of the medium from hypotonic (0.5 M NaCl) to hypertonic (3 M NaCl). Thus, other mechanisms, such as protein modifications, might be involved in the response to changes in osmotic pressure [24].

### Mycotoxin gene cluster analysis and mycotoxin detection

Mycotoxins are important because they can affect human health [25, 43]. Mycotoxins are produced by various filamentous ascomycetes, especially members of the genera *Aspergillus* and *Fusarium*, through well-defined biosynthetic pathways. This issue is relevant to *A. cristatus* because of its ‘generally recognised as safe’ status and its use in the production of Fuzhuan brick tea. The biosynthetic genes and pathways for six mycotoxins (aflatoxin, fumonisin, gliotoxin, ochratoxin, sterigmatocystin and zearalenone) were searched in the genome [15–20]. *Aspergillus cristatus* contains 39 secondary metabolite gene clusters (Table 3). Several backbone enzymes of the mycotoxin gene clusters, which were predicted by antiSMASH [44], are orthologs of the backbone enzymes of some mycotoxin gene clusters (Table 3). The genome contains 14 NRPS- and 14 PKS-encoding genes, most of which are located in clusters. As shown in Additional file 1: Table S3, 85 potential homologs of mycotoxin biosynthetic genes were found in the *A. cristatus* genome using BLASTp and InParanoid 7.0 [45]. It should be noted that in all cases, the homologs of mycotoxin biosynthetic genes are not located in mycotoxin gene clusters (Additional file 1: Table S3). None of the predicted gene clusters showed collinearity with known mycotoxin gene clusters. Thus, there are no known mycotoxin biosynthetic gene clusters in the *A. cristatus* genome.

**Table 3:** Secondary metabolism gene clusters in *Aspergillus cristatus*

Cluster	Scafflod	Backbone enzymes	Genes	Predicted products
Cluster1	1	PKS	SI65_01032-01050	stigmatellin
Cluster2	1	Lanthionine	SI65_01066-01075	
Cluster3	1	Siderophore	SI65_01345-01350	
Cluster4	1	Terpene	SI65_01890-01900	
Cluster5	1	Terpene	SI65_01982-01992	
Cluster6	2	PKS	SI65_02155-02174	hypothemycin
Cluster7	2	PKS	SI65_02372-02384	
Cluster8	2	NRPS	SI65_02625-02642	
Cluster9	2	PKS	SI65_02638-02656	
Cluster10	2	PKS	SI65_02843-02864	
Cluster11	3	PKS	SI65_03265-03285	
Cluster12	3	PKS	SI65_03715-03729	
Cluster13	3	NRPS	SI65_03775-03795	
Cluster14	3	PKS	SI65_03940-03953	
Cluster15	4	NRPS	SI65_04496-04511	
Cluster16	4	NRPS	SI65_05048-05065	
Cluster17	5	NRPS	SI65_05225-05238	
Cluster18	5	NRPS	SI65_05406-05426	
Cluster19	5	PKS	SI65_05759-05776	
Cluster20	6	NRPS	SI65_06121-06138	
Cluster21	7	PKS	SI65_06667-06682	
Cluster22	8	NRPS	SI65_07091-07104	
Cluster23	8	Terpene	SI65_07245-07259	
Cluster24	9	PKS	SI65_07348-07363	
Cluster25	9	Lanthionine	SI65_07571-07579	
Cluster26	10	Terpene	SI65_07672-07681	
Cluster27	11	Siderophore	SI65_07931-07938	
Cluster28	12	NRPS	SI65_08199-08214	
Cluster29	12	NRPS	SI65_08311-08325	
Cluster30	13	NRPS	SI65_08399-08408	
Cluster31	13	NRPS	SI65_08558-08578	
Cluster32	13	PKS	SI65_08470-08482	
Cluster33	14	Terpene	SI65_08621-08631	
Cluster34	21	PKS	SI65_09731-09746	
Cluster35	21	Terpene	SI65_09793-09801	
Cluster36	22	PKS	SI65_09879-09899	
Cluster37	24	Terpene	SI65_10001-10023	
Cluster38	25	NRPS	SI65_10088-10103	
Cluster39	26	NRPS	SI65_10253-10268	

In general, the biosynthesis genes for fungal secondary metabolites are located in clusters [21]; however, the mycotoxin genes in *A. crastatus* were not found located in clusters. Moreover, transcription data showed that most of the mycotoxin genes are expressed at low and high osmotic pressure (Additional file 1: Table S3). This result suggested that *A. crastatus* might produce mycotoxins during culture under low and high osmolarity conditions. To confirm this, we assayed six mycotoxins by High Performance Liquid Chromatography (HPLC). Only fumonisin B1 was detected at 0.17 and 0.15 ppm at low and high osmolarities, respectively. However, this is lower than the standard (2 ppm) set by the US Food and Drug Administration (FDA) [46] (Additional file 2: Figure S2). These data suggest that the strain is safe under low- and high-osmolarity conditions, and the locations of the mycotoxin genes did not provide information regarding mycotoxin production by the fungus.

## Conclusions

Comparison of the *MAT* loci of *A. cristatus* with those of other *Aspergillus* species revealed that the evolution of the *A. cristatus MAT* locus differs from those of other *Aspergillus* species. The findings regarding the *Aspergillus MAT* loci supported the evolution of homothallism from heterothallism. The majority of sex-related components identified in other ascomycetes are also present in *A. cristatus*. The initial analysis of the evolution of the *MAT* loci, associated with sex-related components, provides information for further investigation of sexual development in *A. cristatus*.

The components of the HOG pathway were conserved in *A. cristatus*. Gene expression analysis demonstrated that the HOG pathway of *A. cristatus* was not involved in the response to high osmotic pressure. Thus, *A. cristatus* may respond to high osmolarity stress via mechanisms other than the HOG pathway.

A mycotoxin gene cluster collinearity analysis indicated that the mycotoxin biosynthetic gene clusters responsible for production of six toxins were not present in the *A. cristatus* genome. An HPLC assay indicated that the strain is safe under low- and high-osmolarity conditions; moreover, the locations of the mycotoxin genes did not provide information regarding mycotoxin production by this fungus.

## Methods

### Strains, growth conditions, and genomic DNA and RNA extraction

*A. cristatus* E4 (CGMCC 7.193) was isolated from Fuzhuan brick tea in a Yiyang Tea Factory in Yi Yang City, Hunan Province, China. The fungus was cultured in liquid medium (malt extract 20 g, yeast extract powder 20 g, sucrose 30 g, and water 1000 mL) with shaking at 180 rpm at 28 °C for 5 days. Mycelia were collected by filtration on Waterman paper and placed in a mortar, liquid nitrogen was added, and the samples were crushed using a pestle. Genomic DNA from fungal mycelia was extracted using the CTAB method [47]. The DNA pellet was dissolved in sterile water and adjusted to a concentration of 500 μg/mL. The fungus was cultured on a cellulose membrane on MYA (malt extract 20 g, yeast extract powder 20 g, sucrose 30 g, agar powder 18 g, and water 1000 mL) plates in the dark at 28 °C for 7 days. Mycelia were collected after sporulation. Total RNA was extracted from fresh mycelia using TRIzol reagent according to the manufacturer’s instructions (Life Technologies Co. Ltd., Carlsbad, CA, USA), and the RNA pellet was dissolved in sterile water containing diethylpyrocarbonate diluted 1:1000 in sterile water. DNA and RNA were quantified using a Nano Drop 2000 UV–vis spectrophotometer (Thermo Fisher Scientific Inc., Boston, MA, USA) based on the absorbance at 260 and 280 nm, respectively. DNA and RNA samples were subjected to genome and transcriptome sequencing by BGI-Shenzhen (Shenzhen, China).

### Sequencing and assembly

From the genomic DNA of *A. cristatus*, 500 bp and 6 kb DNA sequencing libraries were constructed using 5 μg and 20 μg DNA [48], respectively. A total of 2,364 and 1,125 Mb reads were generated by an Illumina Hiseq™ 2000 at BGI-Shenzhen (Shenzhen, China). To ensure the accuracy of the assembly, reads with 36 low-quality (≤ Q2) bases, 9 % Ns, or 3-bp overlaps between the adapter and duplications were filtered. The short reads from the two libraries were assembled using *SOAPdenovo* 1.04 [49, 50], with optimal assembly acquired using the key parameter *K* = 55.

### Gene prediction and annotation

Gene models were predicted independently using a set of gene finders including Augustus [51], GeneMark-ES [52], GeneId 1.2 [53] and Fgenesh + [54]. Augustus parameters were trained on gene models in *Aspergillus* (*A. fumigatus*, *A. nidulans* and *A. oryzae*) using the transcriptomic data as hints. GeneMark-ES functions in a self-training manner. The available fungal genome sequence of *A. nidulans* was used for the GeneId gene predictor. The predicted gene models were then combined into consensus gene structure annotations using EvidenceModeler [55].

BLASTp searches against the UniProt/SwissProt, KEGG and COG databases were performed to assign general protein function profiles [56–58]. Pfam was used to scan for significant domains using HMMER [59, 60]. Blast2go was used for Gene Ontology (GO) and InterPro annotation [61–63]. Predicted proteins were classified as proteases by querying the MEROPS database using BLASTp (*E*-value cut-off of 1e-10) [64]. Potential secondary metabolite key enzyme genes were identified using antiSMASH 1.2.2 [44]. Transposons and retrotransposons encoding transposases and retrotransposases were classified by BLASTp analysis against the Repbase database [65].

### Orthology and phylogenetic analysis

Orthologous groups were clustered using the OrthoMCL version 2.0 software with an *E*-value cut-off of 1e-5 and percentage match cut-off of 50 [66, 67]. In total, 1,034 single-copy orthologous proteins were acquired and aligned using MAFFT 7.221 [68]. A maximum-likelihood phylogenetic tree was created using the concatenated amino acid sequences and the RaxML 7.2.8 software [69], with the best model, which was simulated using the ProtTest 1.4 software [70].

### Transcriptome analysis

Raw data generated by the sequencer were converted to raw nucleotide reads by Illumina GAPipeline 1.6. Clean reads were acquired by removing the adaptor and the low-quality reads (Q ≤ 5), and were mapped to the genome using Tophat [71]. Up to two base mismatches were allowed. The abundance of each clean read was converted to transcripts per million for quantitative comparison among samples. We used the false discovery rate (FDR ≤0.001) to estimate the level of differential gene expression among samples under different induction conditions [72]. Genes with FDR values less than 0.001 and log2-fold changes greater than 2.0 or lower than −2.0 were considered to be differentially expressed.

### Mycotoxin gene clusters analysis and mycotoxin detection

The mycotoxin gene clusters in this study were obtained from GenBank (Additional file 1: Table S3), and their sequences applied as BLAST queries against the protein sequences of *A. cristatus* (*E*-value < 1e-5). A homology analysis was performed using InParanoid 7.0 (*E*-value < 0.01, score >50) [45]. To detect mycotoxins in the end products, mycelia were incubated in the dark at 28 °C for 9 days with 0.5 M or 3.0 M NaCl and collected on cellulose membranes after sporulation. Mycelia were processed using various methods [73–78] and then analysed by HPLC (Hitachi L-2000).

### Availability of data

All data contributing to this genome initiative has been deposited at the NCBI under BioProject PRJNA271918; the genome accession number is [JXNT00000000]. The genome version described in this paper is the first version and the accession number is [JXNT01000000]. The RNA-seq expression dataset has been deposited at the NCBI’s Gene Expression Omnibus under the accession code GSE65662. The phylogenetic analysis results are deposited in TreeBase (http://purl.org/phylo/treebase/phylows/study/TB2:S19105).

### Ethics statement

No specific permissions were required for these locations/activities. The field studies did not involve endangered or protected species and were conducted in accordance with local legislation.

## Additional files

Additional file 1: Table S1.
*Aspergillus cristatus* genome statistics compared to that of other sequenced *Aspergillus* fungi. **Table S2.** Genes used for Phylogenetic Analysis. **Table S3.** Homology Genes of Mycotoxin Biosynthesis in *Aspergillus cristatus* Genome. (PDF 808 kb) Additional file 2: Figure S1. Results of *MAT1-1-1* and *MAT1-2-1* amino acid sequence analysis of members of the genus *Aspergillus* using Jalview version 2.0 [32]. **Figure S2.** Detection of six mycotoxins by HPLC. (PDF 401 kb)

## Abbreviations

FDA: Food and drug administration
FPKM: Fragments per kilobase of exon per million fragments mapped
HMG: High-mobility group
HOG: The high-osmolarity glycerol
HPLC: High performance liquid chromatography
MAT: Mating-typing gene
MAPK: Mitogen-activated oprotein kinase
MAPKK: MAPK kinase
MAPKKK: MAPKK kinase
