# Supplementary material for: Comparative genomic and transcriptomic analyses of the Fuzhuan brick tea-fermentation fungus Aspergillus cristatus
Source: BMC Genomics. 2016 Jun 7;17:428. doi: 10.1186/s12864-016-2637-y (PMC4895823; doi:10.1186/s12864-016-2637-y)
Supplement: Additional file 1: Table S1. — Aspergillus cristatus genome statistics compared to that of other sequenced Aspergillus fungi. Table S2. Genes used for Phylogenetic Analysis. Table S3. Homology Genes of Mycotoxin Biosynthesis in Aspergillus cristatus Genome. (PDF 808 kb) [file 12864_2016_2637_MOESM1_ESM.pdf]

Additional files 2

Table S1 *Aspergillus cristatus* Genome Statistics compared to that of other sequenced *Aspergillus* Fungi

Genome name	Genome specifics	Length (Mp)	Number of Scaffolds	N50（bp）	Protein-coding	GC content (%)	Gene density (1 gene every n bp)
A. clavatus	NRRL 1	27.85	143	2,493,640	9125	49.2	2940
A. cristatus	**CGMCC 7.193**	**27.9**	**68**	**2,308,221**	**10136**	**49.7**	**2980**
A. flavus	NRRL3357	39.91	331	2,388,123	13485	48.4	2959
A. fumigatus	Af293	29.42	19	2,460,113	9969	49.8	2938
A. kawachii	IFO4308	36.88	318	897,000	11488	49	3210
A. nidulans	FGSC A4	30.06	89	2,440,000	9541	50.4	3151
A. niger	CBS 513.88	35.74	20	2,525,243	11408	50.4	3133
A. oryae	3.042	36.54	225	395,582	11397	48.3	3206
A. parasiticus	SU-1	39.47	753	140,138	8645	48.1	4566
A. rambellii	SRRC1468	26.45	4177	37,599	7761	45.8	3408
A. ruber	CBS 135680	26.21	110	623,674	10076	48.8	2601
A. sojae	NBRC 4239	39.5	65	2,061,651	13033	48.1	3031
A. terreus	NIH 2624	29.23	27	1,912,493	10551	52.9	2770
N. fischeri	NRRL 181	32.54	438	2,929,192	10729	49.4	3033

The data is from the genomic data published online (http://www.ncbi.nlm.nih.gov).

Table S2 Genes used for Phylogenetic Analysis

Gene number	Function description
SI65_00047	UDP-Glycosyltransferase/glycogen_phosphorylase
SI65_00048	Cation_efflux_protein
SI65_00082	DNA_topoisomerase
SI65_00103	Ribosome_maturation_protein_SBDS
SI65_00105	Ribosomal_protein_S13
SI65_00135	Ubiquitin
SI65_00136	Cleavage/polyadenylation_specificity_factor
SI65_00140	U3_small_nucleolar_RNA-associated_protein_10
SI65_00217	TFIIH_p62_subunit
SI65_00241	Uncharacterised_protein_family,_ATP_binding
SI65_00249	Cytoskeleton-associated_protein
SI65_00250	transition_metal_ion_binding
SI65_00252	Mitochondrial_import_inner_membrane_translocase_subunit_Tim54
SI65_00253	NARP1
SI65_00257	Iron_hydrogenase,_large_subunit,_C-terminal
SI65_00259	Glycosyl_transferase,_family_3
SI65_00265	Vacuolar_protein_sorting-associated_protein_26
SI65_00266	Uncharacterized_conserved_protein
SI65_00267	Ubiquitin-conjugating_enzyme,_E2
SI65_00269	RWD_domain
SI65_00272	Putative_RNA_methylase
SI65_00275	Ribosomal_Proteins_L2,_RNA_binding_domain
SI65_00283	Ribonuclease_II/R
SI65_00290	Mitochondrial_substrate/solute_carrier
SI65_00296	Cell_differentiation,_Rcd1-like
SI65_00299	GINS_complex
SI65_00300	Sulphate_transporter
SI65_00303	Asparagine_synthase
SI65_00304	Vacuolar_sorting_protein_9
SI65_00314	ATP-grasp_fold,_ATP-dependent_carboxylate-amine_ligase-type
SI65_00318	Regulator_of_G_protein_signalling
SI65_00328	Leucine-rich_repeat Endonuclease
SI65_00333	ribosome_biogenesis
SI65_00335	Class_II_aldolase
SI65_00340	GPI_mannosyltransferase
SI65_00344	Monooxygenase,_FAD-binding
SI65_00348	Proprotein_convertase
SI65_00353	SNARE_associated_Golgi_protein
SI65_00389	ORMDL
SI65_00404	Ribosomal_protein_S24

SI65_00416	Ovarian_tumour,_otubain
SI65_00423	CDP-alcohol_phosphatidyltransferase
SI65_00431	Protein_of_unknown_function_DUF2370
SI65_00440	WD40_repeat,_subgroup
SI65_00442	Autophagy-related_protein_9
SI65_00466	Conserved_oligomeric_complex_COG6
SI65_00478	Aminoacyl-tRNA_synthetase
SI65_00483	Anthranilate_synthase_component_I
SI65_00497	Domain_of_unknown_function_DUF2427
SI65_00503	Gaa1-like,_GPI_transamidase_component
SI65_00504	Proteasome_component_(PCI)_domain
SI65_00513	HEAT Protein_of_unknown_function_DUF3434
SI65_00522	Armadillo-type_fold
SI65_00531	HAD-like_domain
SI65_00534	Transcription_factor_TFIIE_beta_subunit,_DNA-binding_domain
SI65_00535	3,4-dihydroxy-2-butanone_4-phosphate_synthase,_RibB
SI65_00541	Autophagy-related,_C-terminal
SI65_00552	Fructose-1,6-bisphosphatase_class_1
SI65_00570	CDC24_calponin
SI65_00575	NAD(P)-binding_Rossmann-fold_domains
SI65_00578	ATPase
SI65_00581	Vacuolar_protein_sorting-associated_protein
SI65_00595	intrinsic_to_membrane
SI65_00622	Serine/threonine-protein_kinase-like_domain
SI65_00625	NGP1
SI65_00638	transferase_activity
SI65_00646	GPI_mannosyltransferase
SI65_00647	Aminotransferase_class-III
SI65_00706	Chaperonin_Cpn60/TCP-1
SI65_00707	Ribosomal_protein_L7/L12,_C-terminal
SI65_00712	YEATS
SI65_00729	Domain_of_unknown_function_DUF2404
SI65_00737	Delayed-early_response_protein/equilibrative_nucleoside_transporter
SI65_00742	Cdc37,_C-terminal // Cdc37,_N-terminal // Cdc37,_Hsp90_binding
SI65_00743	Clathrin_light_chain
SI65_00758	Homeobox
SI65_00763	Signal_transduction_histidine_kinase,_phosphotransfer_(Hpt)_domain
SI65_00770	Prenyltransferase/squalene_oxidase
SI65_00787	SART-1_protein
SI65_00788	Peptidyl-tRNA_hydrolase,_PTH2
SI65_00794	Pyridoxal_phosphate-dependent_decarboxylase
SI65_00796	Exocyst_complex_component_Sec6
SI65_00798	Frataxin-like
SI65_00806	Protein_prenyltransferase,_alpha_subunit

SI65_00823	Ribosomal_protein_S6e
SI65_00827	3-oxo-5-alpha-steroid_4-dehydrogenase,_C-terminal
SI65_00831	Ribosomal_protein_L28
SI65_00836	FeS_cluster_biogenesis
SI65_00844	Snf7
SI65_00864	MgsA_AAA+_ATPase
SI65_00865	Aminotransferase,_class_I/classII
SI65_00877	Cdk-activating_kinase_assembly_factor_MAT1,_centre
SI65_00879	Aldose_1-epimerase
SI65_00891	Proteasome/cyclosome,_regulatory_subunit
SI65_00897	WD40_repeat,_subgroup
SI65_00914	Ribophorin_II
SI65_00958	tRNA_synthetase
SI65_00959	HEAT
SI65_00993	F-box_domain,_cyclin-like
SI65_01023	Lumazine-binding_protein
SI65_01026	Alpha-D-phosphohexomutase,_alpha/beta/alpha_domain_I
SI65_01031	ATPase,_AAA-type,_core
SI65_01040	Golgi_phosphoprotein_3
SI65_01048	Ribosomal_protein_S2
SI65_01066	GAT
SI65_01074	Ribosomal_protein_S26e
SI65_01076	HEAT // 26S_proteasome_non-ATPase_regulatory_subunit_5
SI65_01080	Uncharacterised_protein_family_UPF0662
SI65_01083	Basic_helix-loop-helix,_Nulp1-type
SI65_01087	Glycosyl_transferase,_family_1
SI65_01154	Domain_of_unknown_function_DUF2431
SI65_01156	Pseudouridine_synthase_II
SI65_01159	eRF1_domain_3 // eRF1_domain_1/Pelota-like // eRF1_domain_2
SI65_01161	Ribosomal_protein_L25
SI65_01165	uridylate_kinase
SI65_01172	D-isomer_specific_2-hydroxyacid_dehydrogenase,_catalytic_domain
SI65_01180	FACT_complex_subunit_Spt16p/Cdc68p
SI65_01188	Uncharacterised_protein_family_UPF0061
SI65_01195	Ribonuclease_HII/HIII
SI65_01196	Mif2
SI65_01198	Methyltransferase_type_11
SI65_01202	Mov34/MPN/PAD-1
SI65_01208	Coproporphyrinogen_III_oxidase,_aerobic
SI65_01209	Light_chain_3_(LC3)
SI65_01210	Mitochondrial_substrate/solute_carrier
SI65_01216	SNARE_interactions_in_vesicular_transport
SI65_01218	Glutamate_synthase
SI65_01224	Protein_of_unknown_function_DUF3639

SI65_01244	Peptidase_C19,_ubiquitin_carboxyl-terminal_hydrolase_2
SI65_01245	Zinc_finger,_C3HC4_RING-type
SI65_01264	Phosphatidate_cytidylyltransferase
SI65_01266	RNA-binding_S4
SI65_01283	Diphthamide_synthesis,_DPH1/DHP2
SI65_01301	WD40_repeat,_subgroup
SI65_01312	Iso_dh
SI65_01317	Pyruvate_kinase
SI65_01364	UbiA_prenyltransferase_family
SI65_01366	Aminoacyl-tRNA_synthetase,_class_II_(D/K/N)
SI65_01370	Aminoacyl-tRNA_synthetase,_class_Ib
SI65_01414	Domain_of_unknown_function_DUF3535
SI65_01429	Inosine/uridine-preferring_nucleoside_hydrolase_domain
SI65_01436	UAA_transporter
SI65_01455	Importin-beta,_N-terminal
SI65_01483	Protein_of_unknown_function_DUF726
SI65_01513	Rab-GAP/TBC_domain
SI65_01517	Vps52/Sac2
SI65_01531	Ribosomal_protein_L27
SI65_01573	Histone_acetyltransferases_subunit_3
SI65_01574	Nitrogen_permease_regulator_2
SI65_01579	Ribosomal_protein_L18a/LX
SI65_01596	Leucine-rich_repeat
SI65_01620	TPR-like
SI65_01621	Sterile_alpha_motif,_type_2
SI65_01626	Uroporphyrinogen_decarboxylase_(URO-D)
SI65_01627	WD40_repeat,_subgroup
SI65_01628	Ribosomal_protein_60S
SI65_01630	Vps53-like,_N-terminal
SI65_01631	TAP, Nuclear_transport_factor_2
SI65_01632	Domain_of_unknown_function_DUF1692
SI65_01636	RNA_polymerase
SI65_01652	sorting_protein_VPS8
SI65_01657	Domain_of_unknown_function_DUF1726
SI65_01668	Ribosomal_protein_L11,_C-terminal_domain // Ribosomal_protein_L11,_N-terminal
SI65_01670	DNA_primase,_large_subunit,_eukaryotic/archaeal
SI65_01672	Staphylococcal_nuclease_(SNase-like)
SI65_01673	Mitochondrial_substrate/solute_carrier
SI65_01674	Peptidase_C2,_calpain,_catalytic_domain
SI65_01678	DNA_topoisomerase_I,_DNA_binding,_eukaryotic-type
SI65_01679	ATPase,_F1_complex,_gamma_subunit
SI65_01700	DeoxyUTP_pyrophosphatase
SI65_01705	Rab-GAP/TBC_domain

SI65_01712	Initiation_factor_eIF-4_gamma,_MA3
SI65_01719	WD40_repeat
SI65_01720	alpha/beta-Hydrolases
SI65_01722	NPL4,_zinc-binding_putative // NPL4
SI65_01724	Zinc_finger,_CCCH-type
SI65_01755	Ribosomal_protein_S24e
SI65_01761	adenyl_ribonucleotide_binding
SI65_01770	Uncharacterized conserved protein
SI65_01775	Clathrin // Clathrin,_heavy_chain,_propeller_repeat
SI65_01819	Aminoacyl-tRNA_synthetase,_class_I_(M)
SI65_01821	Protein_prenyltransferase,_alpha_subunit
SI65_01828	Hypoxia_induced_protein,_domain
SI65_01829	Pyridoxal_phosphate-dependent_enzyme,_beta_subunit
SI65_01866	Proteasome,_subunit_alpha/beta
SI65_01881	WD40_repeat,_subgroup
SI65_01883	Peptidase_C19,_ubiquitin_carboxyl-terminal_hydrolase_2
SI65_01885	WD40_repeat,_subgroup // Small-subunit_processome,_Utp13
SI65_01889	Protein_synthesis_factor,_GTP-binding
SI65_01988	Squalene/phytoene_synthase
SI65_01992	Peptidase_M48
SI65_01994	WD40_repeat,_subgroup
SI65_02019	WD40_repeat,_subgroup
SI65_02027	UPF0103/Mediator_of_ErbB2-driven_cell_motility_(Memo-related)
SI65_02041	Kinetochore_Spc7
SI65_02066	DNA_polymerase_alpha/epsilon,_subunit_B
SI65_02082	Mitochondrial_inner_membrane_protein_Mitofilin
SI65_02086	Myristoyl-CoA:protein_N-myristoyltransferase
SI65_02099	Ribosomal_protein_L5
SI65_02115	WD40_repeat,_subgroup
SI65_02124	UTP--glucose-1-phosphate_uridylyltransferase
SI65_02128	Nicotinamide_N-methyltransferase,_putative
SI65_02153	tRNA-dihydrouridine_synthase
SI65_02193	Ribulose-phosphate_3-epimerase
SI65_02202	Ssl1-like
SI65_02210	Serine/threonine-protein_kinase-like_domain
SI65_02229	TAFII55_protein,_conserved_region
SI65_02234	Oxoglutarate/iron-dependent_oxygenase
SI65_02241	Type_I_phosphodiesterase
SI65_02246	CCAAT-binding_transcription_factor,_subunit_B
SI65_02329	Histone_deacetylase_interacting // Paired_amphipathic_helix
SI65_02330	WD40_repeat,_subgroup
SI65_02336	Transcription_initiation_factor_IIF,_beta_subunit
SI65_02341	ATPase,_F0_complex,_subunit_B,_mitochondrial
SI65_02342	CCAAT-binding_factor

SI65_02347	MMS19_N
SI65_02349	Protein_of_unknown_function_DUF106,_transmembrane
SI65_02351	Tetrahydrofolate_dehydrogenase/cyclohydrolase
SI65_02352	ATPase,_F1/V1/A1_complex,_alpha/beta_subunit
SI65_02362	Glycine_cleavage_system_P-protein,_N-terminal
SI65_02405	EAP30
SI65_02427	PHP,_C-terminal
SI65_02433	Vacuolar_protein_sorting_protein,_Vps36 // EAP30
SI65_02435	Phox_homologous_domain // Phox/Bem1p // Src_homology-3_domain
SI65_02458	ATPase,_AFG1-like
SI65_02463	Alanine_racemase,_N-terminal
SI65_02472	Transcription_factor_Tfb2
SI65_02474	Timeless_protein // Timeless_C-terminal
SI65_02477	Actin-like
SI65_02491	ATP-citrate_lyase/succinyl-CoA_ligase
SI65_02503	Bacterial_surface_antigen_(D15)
SI65_02509	Sodium/calcium_exchanger_membrane_region
SI65_02510	Carbohydrate_kinase,_FGGY,_C-terminal
SI65_02515	Nucleoporin_interacting_component_Nup93/Nic96
SI65_02536	Exportin-1/Importin-beta-like
SI65_02552	WD40_repeat,_subgroup
SI65_02553	Peptidase_S10,_serine_carboxypeptidase
SI65_02556	ICE2
SI65_02557	Metal-dependent_protein_hydrolase
SI65_02560	Aminoacyl-tRNA_synthetase,_class_I_(M)
SI65_02563	Sorting_nexin
SI65_02564	Phospholipid_methyltransferase
SI65_02577	BOP1,_N-terminal // WD40_repeat,_subgroup
SI65_02587	Cytidylyltransferase
SI65_02589	Digestive_organ_expansion_factor,_predicted
SI65_02597	Ribosomal_protein_L17
SI65_02600	Ribosomal_protein_S12/S23
SI65_02601	Initiation_factor_2B-related
SI65_02603	Aminotransferase,_class_V/Cysteine_desulfurase
SI65_02604	Metallo-dependent_phosphatases
SI65_02613	WD40_repeat,_subgroup
SI65_02619	Serine/threonine-protein_kinase-like_domain
SI65_02620	LsmAD_domain
SI65_02626	RNA_ligase,_T4_RnlA-like
SI65_02641	Nitrogen_permease_regulator_3
SI65_02654	Membrane_transporter,_Tim44-related/Ribosomal_protein_L45
SI65_02676	S-adenosyl-L-homocysteine_hydrolase,_NAD_binding
SI65_02679	NOSIC
SI65_02680	RNA-processing_protein,_HAT_helix

SI65_02696	Phosphoglycerate_kinase
SI65_02705	CPL
SI65_02711	ATPase_assembly_factor_ATP10,_mitochondria
SI65_02712	V-SNARE_C
SI65_02713	Ribosomal_protein_L29
SI65_02738	Glycosyl_transferase,_family_1
SI65_02739	DNA/RNA_helicase
SI65_02745	Fatty_acid_hydroxylase
SI65_02746	Translationally_controlled_tumour_protein
SI65_02751	Beta-Casp_domain
SI65_02755	Aminoacyl-tRNA_synthetase,_class_Ib
SI65_02767	Pseudouridine_synthase,_TruD
SI65_02775	Trimeric_LpxA-like
SI65_02781	Craniofacial_development_protein_1
SI65_02785	Amidohydrolase_2
SI65_02791	ATPase,_V1_complex,_subunit_C
SI65_02795	Glycine_cleavage_T-protein
SI65_02802	WD40_repeat,_subgroup
SI65_02805	ATPase,_V1_complex,_subunit_H
SI65_02810	S-adenosylmethionine_synthetase,_C-terminal
SI65_02825	Nucleolar,_Nop52
SI65_02827	Clathrin/coatomer_adaptor,_adaptin-like
SI65_02880	Glutamate-cysteine_ligase_catalytic_subunit
SI65_02912	ATPase,_AAA-type,_VAT
SI65_02915	Mo25-like
SI65_02919	Ankyrin_repeat
SI65_02938	Dual_specificity_phosphatase,_catalytic_domain
SI65_02952	Cyclin,_N-terminal
SI65_02965	Phox_homologous_domain
SI65_02968	THO_complex,_subunitTHOC2
SI65_02975	Serine/threonine-protein_kinase-like_domain
SI65_02976	RNA_recognition_motif_domain
SI65_02978	Carbamoyl-phosphate_synthase,_small_subunit
SI65_02981	PIG-X
SI65_03006	DNA_repair_protein_rad10
SI65_03017	Serine/threonine-protein_kinase-like_domain
SI65_03023	WD40_repeat,_subgroup
SI65_03034	Domain_of_unknown_function_DUF1279
SI65_03035	Ribosomal_protein_L3
SI65_03041	PRELI/MSF1
SI65_03046	Mpp10_protein
SI65_03049	Nucleoporin,_Nup133/Nup155-like
SI65_03058	Glutamine_amidotransferase,_class-II
SI65_03064	methyltransferase,_Williams-Beuren_syndrome

SI65_03067	Mitochondrial_distribution_and_morphology_protein_family_31/32
SI65_03068	K_Homology,_type_1,_subgroup
SI65_03069	Proteasome,_subunit_alpha/beta
SI65_03071	Proteasome,_subunit_alpha/beta
SI65_03077	WD40_repeat,_subgroup
SI65_03084	Eukaryotic_phosphomannomutase
SI65_03094	Sybindin-like_protein
SI65_03098	Translation_initiation_factor_IF6
SI65_03102	Ubiquitin
SI65_03108	Domain_of_unknown_function_DUF2427
SI65_03109	UBA/THIF-type_NAD/FAD_binding_fold
SI65_03112	ATP-citrate_lyase
SI65_03113	GCN5-related_N-acetyltransferase_(GNAT)_domain
SI65_03114	Histidine_biosynthesis
SI65_03116	Zinc_finger,_C2H2-type
SI65_03120	Aminotransferase
SI65_03121	RNA_polymerase_I_associated_factor,_A49-like
SI65_03124	Peroxin-3
SI65_03130	Ribosomal_Proteins_L2,_RNA_binding_domain
SI65_03133	Carbohydrate/purine_kinase
SI65_03138	Ribosomal_protein_L5
SI65_03141	Clathrin
SI65_03144	Sad1/UNC-like
SI65_03149	GTP-binding_domain,_HSR1-related
SI65_03150	Low_temperature_viability_protein
SI65_03151	Phospholipase_D/nuclease
SI65_03200	Mur_ligase,_central
SI65_03207	HhH-GPD_domain
SI65_03216	Asparagine_synthase
SI65_03223	Rad52/22_double-strand_break_repair_protein
SI65_03225	Amino_acid_transporter,_transmembrane
SI65_03240	Cytochrome_c/c1_haem-lyase
SI65_03241	GHMP_kinase
SI65_03242	IKI3
SI65_03247	Aspartate/ornithine_carbamoyltransferase
SI65_03249	Acetolactate_synthase,_small_subunit
SI65_03255	Mur_ligase,_central
SI65_03346	organellar_small_ribosomal_subunit
SI65_03347	Signal_recognition_particle_receptor,_alpha_subunit
SI65_03349	Hrf1
SI65_03352	ATPase,_F0_complex,_subunit_D,_mitochondrial
SI65_03354	Aldehyde_dehydrogenase_domain
SI65_03359	Glutamine_amidotransferase,_class-II
SI65_03382	Protein_of_unknown_function_DUF3548

SI65_03383	Protein_of_unknown_function_DUF3402
SI65_03384	Phosphatidic_acid_phosphatase_type_2
SI65_03397	RWD_domain
SI65_03398	Armadillo-type_fold
SI65_03403	D123
SI65_03408	CAS/CSE
SI65_03421	DNA/RNA_helicase
SI65_03434	CDP-alcohol_phosphatidyltransferase
SI65_03435	Glucose-inhibited_division_protein_A-related
SI65_03436	Acireductone_dioxygenase_ARD_family
SI65_03439	Homeodomain-like
SI65_03440	E3_ubiquitin_ligase
SI65_03442	ABC-1
SI65_03446	Peptidase_M22,_glycoprotease
SI65_03447	SET_domain
SI65_03448	Enhancer_of_polycomb-like,_N-terminal
SI65_03451	Ribosomal_protein_S9
SI65_03454	Protein_of_unknown_function_DUF947
SI65_03466	Imidazoleglycerol-phosphate_dehydratase
SI65_03469	Mitochondrial_protein_Pet127
SI65_03472	Nucleotide_exchange_factor_Fes1
SI65_03473	Uncharacterised_protein_family_UPF0220
SI65_03481	Transport_protein_Trs120
SI65_03484	Membrane_bound_O-acyl_transferase,_MBOAT
SI65_03486	Abortive_infection_protein
SI65_03498	Aconitase_A
SI65_03506	Uncharacterised_protein_family_UPF0363
SI65_03508	tRNA_isopentenyltransferase
SI65_03509	P-loop_containing_nucleoside_triphosphate_hydrolases
SI65_03513	Porin,_eukaryotic_type
SI65_03515	MIF4G-like,_type_2
SI65_03531	Exocyst_complex_subunit_Sec15-like
SI65_03554	TIP41-like_protein
SI65_03561	Pyridoxal_phosphate-dependent_enzyme
SI65_03568	Serine/threonine-protein_kinase-like_domain
SI65_03623	RNA-binding_S4
SI65_03627	Orotidine_5'-phosphate_decarboxylase_domain
SI65_03631	WD40_repeat,_subgroup
SI65_03636	TFIIE_alpha
SI65_03638	Domain_of_unknown_function_DUF1709
SI65_03645	Peptidyl-prolyl_cis-trans_isomerase
SI65_03649	RNA_recognition_motif_domain
SI65_03653	Phospholipid/glycerol_acyltransferase
SI65_03660	Zinc_finger,_C3HC4_RING-type

SI65_03662	DNA/RNA_non-specific_endonuclease
SI65_03668	Carbamoyl-phosphate_synthetase
SI65_03690	Exoribonuclease,_phosphorolytic_domain_2
SI65_03693	Dihydrofolate_reductase_domain
SI65_03713	Ankyrin_repeat-containing_domain
SI65_03727	DNA/RNA_helicase,_DEAD/DEAH_box_type
SI65_03739	RIO-like_kinase
SI65_03742	F-box_domain,_cyclin-like
SI65_03753	Mitochondrial_inner_membrane_translocase_complex
SI65_03773	WD40_repeat,_subgroup
SI65_03805	UBA/THIF-type_NAD/FAD_binding_fold
SI65_03814	Nse4
SI65_03815	Phosphatidylinositol_3-/4-kinase
SI65_03841	Oligosaccharyl_transferase_complex,_subunit_Wbp1
SI65_03845	DNA_polymerase
SI65_03854	WD40_repeat,_subgroup
SI65_03872	Domain_of_unknown_function_DUF1620
SI65_03877	BEACH_domain
SI65_03887	Inhibitor_of_apoptosis-promoting_Bax1-related
SI65_03912	Histone_H3-K56_acetyltransferase,_RTT109
SI65_03932	Glycoside_hydrolase,_family_31
SI65_03933	Nucleoside_diphosphate_kinase
SI65_03941	PLU-1-like
SI65_03950	Zinc_ribbon,_NADH_pyrophosphatase
SI65_03951	Nitrilase
SI65_03954	Helicase
SI65_03962	GOLD
SI65_03976	WD40_repeat,_subgroup
SI65_03980	SKI-interacting_protein_SKIP
SI65_03983	Ribosomal_protein_L4/L1e
SI65_04021	Splicing_factor_3B_subunit_1
SI65_04022	Sec1-like_protein
SI65_04026	Telomere_length_regulation_protein,_conserved_domain
SI65_04062	Ribonuclease_P
SI65_04070	Ubiquitin
SI65_04072	WD40_repeat,_subgroup
SI65_04080	BRCT
SI65_04083	Restriction_endonuclease
SI65_04089	AdoMet-dependent_methyltransferase,_predicted
SI65_04093	DNA/RNA_helicase
SI65_04097	Serine/threonine-protein_kinase-like_domain
SI65_04100	P-loop_containing_nucleoside_triphosphate_hydrolases
SI65_04111	Phospholipase/carboxylesterase
SI65_04159	Chaperonin_Cpn60

SI65_04187	Mitochondrial_substrater
SI65_04188	F-box_domain,_Skp2-like
SI65_04194	AP_complex
SI65_04196	SUA5
SI65_04200	Hydroxyethylthiazole_kinase
SI65_04203	eIF4-gamma/eIF5/eIF2-epsilon
SI65_04206	Armadillo-type_fold
SI65_04240	Cytochrome_c_oxidase,_subunit_Va
SI65_04243	AIR_synthase-related_protein
SI65_04252	AMP-dependent_synthetase/ligase
SI65_04254	CWC16_protein
SI65_04255	Phosphatidylinositol_3-/4-kinase
SI65_04275	Polyprenyl_synthetase
SI65_04278	Zinc_finger,_double-stranded_RNA_binding
SI65_04280	WD40_repeat,_subgroup
SI65_04282	ATPase,_F1/V1/A1_complex,_alpha/beta_subunit
SI65_04303	Armadillo-type_fold
SI65_04311	Dephospho-CoA_kinase
SI65_04338	Arb2_domain // Histone_deacetylase_domain
SI65_04340	Inorganic_phosphate_transporter_Pho88
SI65_04343	Single-stranded_nucleic_acid_binding_R3H
SI65_04367	Zinc_finger,_ZPR1-type
SI65_04369	Metallo-dependent_phosphatase
SI65_04372	Leucine-rich_repeat
SI65_04374	Clathrin // VPS11_C
SI65_04407	Ras
SI65_04408	WD40_repeat,_subgroup
SI65_04421	Sterile_alpha_motif,_type_2
SI65_04424	Domain_of_unknown_function_DUF298
SI65_04444	Mitochondrial_substrate/solute_carrier
SI65_04457	SNF5/SMARCB1/INI1
SI65_04462	Peptidase_M24,_structural_domain
SI65_04465	Glycosyl_transferase,_ALG6/ALG8
SI65_04467	Mitochondrial_substrate/solute_carrier
SI65_04469	NMD3
SI65_04475	Snf7
SI65_04478	Poly_A_polymerase,_head_domain
SI65_04485	DNA-directed_DNA_polymerase,_family_B,_exonuclease_domain
SI65_04512	Oxidoreductase
SI65_04521	Glutamyl/glutaminyl-tRNA_synthetase,_class_Ic,_catalytic_domain
SI65_04522	Microtubule-associated_protein,_MAP65/ASE1-type
SI65_04548	Histidine_triad_(HIT)_protein
SI65_04552	Importin-alpha-like,_importin-beta-binding_domain
SI65_04553	STAG

SI65_04557	Ribosomal_protein_S1,_RNA-binding_domain
SI65_04560	Aminoacyl-tRNA_synthetase
SI65_04564	N-methyltransferase_activity
SI65_04572	Importin-beta,_N-terminal
SI65_04587	Cyclin,_N-terminal
SI65_04588	EF-hand
SI65_04621	Patatin
SI65_04625	Actin-like
SI65_04627	YL1_nuclear,_C-terminal
SI65_04654	Serine/threonine-protein_kinase-like_domain
SI65_04656	Bud13
SI65_04717	Heat_shock_protein_DnaJ,_N-terminal
SI65_04719	RNA_recognition_motif_domain
SI65_04740	Endoplasmic_reticulum_oxidoreductin_1
SI65_04763	Leukotriene_A4_hydrolase
SI65_04777	RNase_P_subunit_p30
SI65_04786	DNA_mismatch_repair_protein_MutS
SI65_04794	Serine/threonine-protein_kinase-like_domain
SI65_04797	RNA_3'-terminal_phosphate_cyclase_domain
SI65_04800	Heat_shock_cognate_protein_B
SI65_04801	Pre-mRNA_processing_factor_4_(PRP4)-like
SI65_04802	Protein_phosphatase_2C-like
SI65_04820	Oligosaccharyl_transferase,_STT3_subunit
SI65_04822	Protein_of_unknown_function_DUF3453
SI65_04833	Predicted_methyltransferase
SI65_04839	ATPase,_AAA-type,_core
SI65_04840	Sec62/63_complex,_subunit_Sec66
SI65_04848	Putative_Zn-finger_protein
SI65_04863	Cleavage/polyadenylation_specificity_factor,_A_subunit,_C-terminal
SI65_04887	Ssl1-like
SI65_04901	Mitochondrial_substrate/solute_carrier
SI65_04902	Peptidase_M16
SI65_04903	RNA_polymerase_III_subunit_RPC82-related,_helix-turn-helix
SI65_04915	CS_domain // SGS
SI65_04923	Ribosomal_protein_L27
SI65_04940	Signal_recognition_particle,_SRP54_subunit,_M-domain
SI65_04967	SEP_domain
SI65_04969	Membrane_insertion_protein,_OxaA/YidC
SI65_04975	S-adenosylmethionine_decarboxylase
SI65_04976	TATA_box_binding_protein_associated_factor_(TAF)
SI65_04977	Cellular_retinaldehyde-binding/triple_function
SI65_05004	Gtr1/RagA_G_protein
SI65_05010	Exportin-1/Importin-beta-like
SI65_05011	Carbohydrate_kinase

SI65_05032	Metallo-dependent_phosphatase
SI65_05034	Mitochondrial_substrate/solute_carrier
SI65_05037	Peptidase_C50,_separase
SI65_05038	Putative_esterase
SI65_05039	Ribosomal_protein_S15
SI65_05042	8-oxoguanine_DNA_glycosylase
SI65_05045	Galactose_oxidase
SI65_05047	Structure-specific_recognition_protein
SI65_05091	Ketopantoate_reductase_ApbA/PanE
SI65_05100	Helicase
SI65_05101	PGAP1-like
SI65_05111	TPR-like
SI65_05116	Metallo-dependent_phosphatase // Mre11,_DNA-binding
SI65_05120	Protein_import_receptor_MAS20-related
SI65_05121	Vps51/Vps67
SI65_05122	ABC_transporter-like
SI65_05138	NUDIX_hydrolase_domain
SI65_05142	Serine/threonine-protein_kinase-like_domain
SI65_05143	Replication_protein_A
SI65_05149	Phosphoadenosine_phosphosulphate_reductase
SI65_05161	Domain_of_unknown_function_DUF2183
SI65_05170	Fry-like_conserved_proteins
SI65_05179	Pre-mRNA_polyadenylation_factor_Fip1
SI65_05187	UBA/THIF-type_NAD/FAD_binding_fold
SI65_05188	Ribose_5-phosphate_isomerase
SI65_05194	tRNA_processing
SI65_05219	Aminoacyl-tRNA_synthetase
SI65_05222	RAVE_protein_1
SI65_05262	Tetrapyrrole_methylase
SI65_05273	DEAD2
SI65_05274	RNA_polymerase,_alpha_subunit
SI65_05275	Pre-mRNA-splicing_factor_3
SI65_05277	GPI_mannosyltransferase
SI65_05298	RNA_polymerase
SI65_05305	Bacterial_Fmu_(Sun)/eukaryotic_nucleolar_NOL1/Nop2p
SI65_05310	CASP,_C-terminal
SI65_05317	Serine/threonine-protein_kinase-like_domain
SI65_05318	UBX
SI65_05330	Heat_shock_protein_70
SI65_05340	D-arabinono-1,4-lactone_oxidase
SI65_05344	Sec63_domain
SI65_05349	GWT1
SI65_05357	organelle_membrane
SI65_05358	B-cell_receptor-associated_31-like

SI65_05359	tRNA-dihydrouridine_synthase
SI65_05360	C2H2_and_C2HC_zinc_fingers
SI65_05369	WD40_repeat,_subgroup
SI65_05371	WD40_repeat,_subgroup
SI65_05376	Transcription_factor_TAFII-31
SI65_05378	Small-subunit_processome,_Utp11
SI65_05379	Phosphoribosyl-AMP_cyclohydrolase
SI65_05389	SDA1
SI65_05390	Ribosomal_protein_S7_domain
SI65_05432	RNA_polymerase_II_subunit_A
SI65_05434	Exoribonuclease,_phosphorolytic_domain_1
SI65_05440	Src_homology-3_domain
SI65_05483	IMP_dehydrogenase/GMP_reductase
SI65_05486	Phenylalanyl-tRNA_synthetase
SI65_05501	Mov34/MPN/PAD-1
SI65_05503	Ribosomal_protein_L14b/L23e
SI65_05537	PROCN
SI65_05539	Cytochrome_c_oxidase,_subunit_Vb
SI65_05546	eRF1_domain_3
SI65_05553	WD40_repeat,_subgroup
SI65_05554	WD40_repeat-like-containing_domain
SI65_05584	rRNA-processing_protein_EFG1
SI65_05595	DNA_topoisomerase,_type_IA
SI65_05603	Ku70/Ku80,_N-terminal_alpha/beta
SI65_05604	Coatomer,_gamma_subunit
SI65_05610	Phosducin
SI65_05631	WD40_repeat,_subgroup
SI65_05633	Exosomal_3'-5'_exoribonuclease_complex_subunit_Rrp40
SI65_05649	UDP-glucose:Glycoprotein_Glucosyltransferase
SI65_05652	Prefoldin_subunit
SI65_05655	phosphotransferase_activity
SI65_05678	Programmed_cell_death_protein_2,_C-terminal
SI65_05682	DNA_polymerase_epsilon,_catalytic_subunit_A
SI65_05685	Chaperonin_Cpn60/TCP-1
SI65_05694	Aminoacyl-tRNA_synthetase,_class_II_(D/K/N)
SI65_05696	Beta-Casp_domain
SI65_05697	Frag1/DRAM/Sfk1
SI65_05715	U3_small_nucleolar_RNA-associated_protein_6
SI65_05726	Helicase/RNase_D_C-terminal,_HRDC_domain
SI65_05727	Lecithin:cholesterol_acyltransferase
SI65_05733	Phosphatidic_acid_phosphatase_type_2/haloperoxidase
SI65_05759	Cyclic_nucleotide-binding_domain
SI65_05761	Electron_transfer_flavoprotein-ubiquinone_oxidoreductase
SI65_05780	TATA-box_binding_protein

SI65_05785	Peroxisome_membrane_anchor_protein_Pex14p,_N-terminal
SI65_05786	CCR4-Not_complex_component,_Not1
SI65_05791	Fps/Fes/Fer/CIP4_homology
SI65_05800	Zinc_finger,_Mcm10/DnaG-type
SI65_05808	Vacuolar_protein_sorting-associated_protein_35
SI65_05822	Nuclear_transport_factor_2
SI65_05830	Transcription_factor_TFIIB,_cyclin-related
SI65_05849	ARP2/3_complex_16kDa_subunit_(p16-Arc)
SI65_05860	GSKIP/TIF31_domain
SI65_05866	Pex19_protein
SI65_05869	Bromodomain
SI65_05871	Suppressor_of_forked
SI65_05872	Lethal_giant_larvae_(Lgl)-like
SI65_05875	THUMP
SI65_05877	Polyprenyl_synthetase
SI65_05886	Armadillo-type_fold
SI65_05900	Clathrin
SI65_05908	ArsA_ATPase
SI65_05909	ATPase,_AAA-type,_core
SI65_05911	Peptidase_A1
SI65_05914	NLI_interacting_factor
SI65_05917	Ureohydrolase
SI65_05949	Glutamyl-tRNA(Gln)_amidotransferase,_subunit_B/E
SI65_05957	Zinc_finger,_C2H2-type
SI65_06071	Zinc_finger,_PHD-finger
SI65_06076	Nicotinate_phosphoribosyltransferase-like
SI65_06091	UTP--glucose-1-phosphate_uridylyltransferase
SI65_06102	Mannosyltransferase,_DXD
SI65_06105	Aminoacyl-tRNA_synthetase,_class_I
SI65_06113	Ribosomal_protein_S5
SI65_06150	Ribonuclease_II/R
SI65_06153	Transport_protein_particle_(TRAPP)_component
SI65_06155	Glycoside_hydrolase,_family_47
SI65_06159	Phosphoribosyltransferase
SI65_06167	Mammalian_uncoordinated_homology_13,_subgroup,_domain_2
SI65_06188	HIT/MYND_zinc_finger-like
SI65_06199	2-oxoacid_dehydrogenase_acyltransferase,_catalytic_domain
SI65_06200	Nrap_protein
SI65_06203	Binding protein
SI65_06204	Armadillo-type_fold
SI65_06205	Serine/threonine-protein_kinase-like_domain
SI65_06207	Ovarian_tumour,_otubain
SI65_06238	Zinc_finger,_CCCH-type
SI65_06252	RNA_degradation

SI65_06255	Ubiquitin_mediated_proteolysis
SI65_06259	Ribosomal_biogenesis,_methyltransferase,_EMG1/NEP1
SI65_06269	Translation_initiation_factor_2A,_beta_propellor-like_domain
SI65_06272	Phosphatidylinositol-4-phosphate_5-kinase,_core
SI65_06274	26S_proteasome_regulatory_subunit
SI65_06278	Domain_of_unknown_function_DUF2404
SI65_06281	Endonuclease
SI65_06284	SAICAR_synthetase
SI65_06289	Mediator_complex,_subunit_Med12
SI65_06294	Fps/Fes/Fer/CIP4_homology
SI65_06295	RNA_recognition_motif_domain
SI65_06297	adenyl_ribonucleotide_binding
SI65_06303	Surfeit_locus_1
SI65_06305	Coenzyme_Q_biosynthesis_Coq4
SI65_06316	GDPGTP_exchange_factor_Sec2p
SI65_06319	Pre-mRNA_splicing_factor_PRP21-like_protein
SI65_06325	Zinc_finger,_DHHC-type,_palmitoyltransferase
SI65_06329	WD40_repeat,_subgroup
SI65_06330	Phosphoadenosine_phosphosulphate_reductase
SI65_06337	Sulphate_adenylyltransferase
SI65_06345	Proteasome_component_(PCI)_domain
SI65_06352	Biotin/lipoate_A/B_protein_ligase
SI65_06353	Pre-mRNA_splicing_Prp18-interacting_factor
SI65_06357	DNA-directed_DNA_polymerase,_family_B,_exonuclease_domain
SI65_06382	Semialdehyde_dehydrogenase
SI65_06400	Uncharacterised_protein_family_UPF0016
SI65_06434	DNA/pantothenate_metabolism_flavoprotein,_C-terminal
SI65_06440	Glycosyl_transferase,_ALG6/ALG8
SI65_06492	Transcription_factor_IIS
SI65_06493	Peptidase_S16
SI65_06499	Chaperone_DnaJ
SI65_06501	Uncharacterised_protein_domain_NUC173
SI65_06513	Ribosomal_protein_L3
SI65_06518	Phosphoglucose_isomerase_(PGI)
SI65_06523	7,8-Dihydro-6-hydroxymethylpterin-pyrophosphokinase,_HPPK
SI65_06540	eIF3g // RNA_recognition_motif_domain
SI65_06543	Ribosomal_protein_S19/S15
SI65_06545	Uncharacterised_protein_family,_carbohydrate_kinase-related
SI65_06592	ATPase,_AAA-type,_core
SI65_06599	Ferredoxin
SI65_06606	UBA/THIF-type_NAD/FAD_binding_fold
SI65_06612	ATPase,_F1/V1/A1_complex,_alpha/beta_subunit
SI65_06613	Mitochondrial_distribution_and_morphology_family_33,_fungi
SI65_06619	Rieske_[2Fe-2S]_iron-sulphur_domain

SI65_06620	GHMP_kinase
SI65_06626	Ribosomal_protein_S12/S23
SI65_06629	Short-chain_dehydrogenase/reductase_SDR
SI65_06631	Helicase
SI65_06644	Ribosome_60S_biogenesis_N-terminal
SI65_06652	L_domain-like
SI65_06653	Pantoate-beta-alanine_ligase
SI65_06658	DNA/RNA_helicase
SI65_06662	Protein_of_unknown_function_DUF775
SI65_06663	tRNA_intron_endonuclease,_catalytic_domain-like
SI65_06664	Ribonuclease_P/MRP,_subunit_p29
SI65_06690	Molybdopterin_binding
SI65_06693	Initiation_factor_2B-related
SI65_06695	Clathrin/coatomer_adaptor,_adaptin-like,_N-terminal
SI65_06697	RHO_protein_GDP_dissociation_inhibitor
SI65_06701	Pre-mRNA_cleavage_complex_II_Clp1
SI65_06703	Inositol_polyphosphate_kinase
SI65_06709	Transcription_initiation_factor_TFIID,_23-30kDa_subunit
SI65_06715	Ribosomal_protein_S5
SI65_06718	Transcription_factor_Tfb4
SI65_06719	Actin-like
SI65_06724	UNC45-central
SI65_06727	Skb1_methyltransferase
SI65_06735	EF-hand
SI65_06744	Proteasome/cyclosome,_regulatory_subunit
SI65_06746	TAP42-like_protein
SI65_06751	Dynein_heavy_chain
SI65_06770	Ribosomal_protein_L11
SI65_06777	Glycosyltransferase,_ALG3
SI65_06778	ATPase,_V1/A1_complex,_subunit_D
SI65_06782	TPR-like
SI65_06785	Histone_methylation_DOT1
SI65_06797	Ras
SI65_06798	Zinc_finger,_MYND-type
SI65_06805	WD40_repeat,_subgroup
SI65_06810	Eukaryotic_rRNA_processing
SI65_06820	Pseudouridine_synthase_II
SI65_06823	Mitochondrial_substrate/solute_carrier
SI65_06825	WD40_repeat,_subgroup
SI65_06845	Transmembrane_receptor,_eukaryota
SI65_06849	Helicase,_C-terminal // SNF2-related // HIP116,_Rad5p_N-terminal
SI65_06850	DNA-directed_DNA_polymerase,_family_A,_palm_domain
SI65_06853	LPPG:FO_2-phospho-L-lactate_transferase_CofD/UPF0052
SI65_06861	Protein_of_unknown_function_DUF3414

SI65_06869	Transport_protein_particle_(TRAPP)_component
SI65_06870	Phosphatidylethanolamine-binding_protein_PEBP
SI65_06871	Ribosomal_protein_L4/L1e
SI65_06876	Crotonase,_core
SI65_06880	Cystathionine_beta-synthase,_core
SI65_06894	DNA_polymerase_alpha,_subunit_B
SI65_06895	Glutamine_amidotransferase_type_1
SI65_06896	intracellular_membrane-bounded_organelle
SI65_06959	Chorismate_mutase,_type_II
SI65_06963	WASH_complex,_F-actin_capping_protein,_alpha_subunit
SI65_06964	HEAT // Importin-beta,_N-terminal
SI65_07002	Patched
SI65_07005	Peptidase_M24B,_X-Pro_dipeptidase/aminopeptidase_P
SI65_07016	Zinc_finger,_DBF-type // Regulatory_subunit_Dfp1/Him1,_central_region
SI65_07026	GAT // VHS
SI65_07027	Gpi16_subunit,_GPI_transamidase_component
SI65_07040	LUC7-related
SI65_07044	DNA/RNA_helicase,_DEAD/DEAH_box_type
SI65_07047	HECT
SI65_07055	UbiE/COQ5_methyltransferase
SI65_07086	Nucleoside_phosphorylase_domain
SI65_07087	Amine_oxidase
SI65_07097	RNA_binding
SI65_07101	DNA_polymerase,_Y-family,_little_finger_domain
SI65_07106	Endonuclease/exonuclease/phosphatase
SI65_07107	Proteasome,_subunit_alpha/beta
SI65_07114	RNA_polymerase_I,_Rpa2_specific
SI65_07115	Cwf19-like_protein,_C-terminal_domain-2
SI65_07119	Down-regulated-in-metastasis_protein
SI65_07129	Rhodanese-like
SI65_07131	Xylose_isomerase,_TIM_barrel_domain
SI65_07152	Domain_of_unknown_function_DUF250
SI65_07164	EF_hand_associated,_type-2
SI65_07169	negative_regulation_of_transcription
SI65_07176	26S_proteasome,_regulatory_subunit_Rpn7
SI65_07180	PEX11
SI65_07183	Phosphoenolpyruvate_carboxykinase,_ATP-utilising
SI65_07185	mRNA_capping_enzyme,_large_subunit
SI65_07205	Heme_A_synthase/Protoheme_IX_farnesyltransferase
SI65_07217	Proteasome,_subunit_alpha/beta
SI65_07226	Ribosomal_protein_S17e
SI65_07230	Protein_of_unknown_function_DUF1077,_TMEM85
SI65_07236	Prephenate_dehydrogenase
SI65_07294	HR1_repeat,_rho-binding

SI65_07314	Fps/Fes/Fer/CIP4_homology
SI65_07316	Myo-inositol-1-phosphate_synthase,_GAPDH-like
SI65_07355	Alcohol_dehydrogenase_GroES-like
SI65_07375	Male_sterility,_NAD-binding
SI65_07376	Mitochondrial_glycoprotein
SI65_07378	DNA_primase,_small_subunit
SI65_07391	Pyridoxal_phosphate-dependent_enzyme,_beta_subunit
SI65_07397	SAC3/GANP/Nin1/mts3/eIF-3_p25
SI65_07402	TPR-lik
SI65_07403	Vacuolar_import/degradation,_Vid27-related
SI65_07462	TLD
SI65_07505	DNA/RNA-binding_protein_Kin17,_conserved_domain
SI65_07506	Bystin
SI65_07510	Concanavalin_A-like_lectin/glucanase
SI65_07513	Transketolase-like,_pyrimidine-binding_domain
SI65_07518	N2,N2-dimethylguanosine_tRNA_methyltransferase
SI65_07526	Uncharacterised_protein_family_UPF0507
SI65_07531	Clathrin/coatomer_adaptor,_adaptin-like,_N-terminal
SI65_07532	Diacylglycerol_acyltransferase
SI65_07544	Kinesin,_motor_domain
SI65_07545	Helicase,_C-terminal
SI65_07551	RNI-like
SI65_07573	Condensin_complex_subunit_2/barren
SI65_07593	Glucosidase_II_beta_subunit-like
SI65_07608	Fps/Fes/Fer/CIP4_homology
SI65_07609	Ribosomal_protein_S23/S29,_mitochondrial
SI65_07611	Ribosomal_biogenesis_regulatory_protein
SI65_07658	Prenyltransferase/squalene_oxidase
SI65_07661	Actin-like
SI65_07662	GTP_cyclohydrolase_II,_RibA
SI65_07666	SAGA-associated_factor_29
SI65_07697	Phospholipase_C,_phosphoinositol-specific,_EF-hand-like
SI65_07704	Late_secretory_pathway_protein_AVL9
SI65_07720	Ribosomal_protein_L18/L5
SI65_07723	Adenylate_cyclase-associated_CAP
SI65_07729	Double-stranded_RNA-binding
SI65_07742	CLASP_N
SI65_07744	Heat_shock_protein_70
SI65_07751	RNA_recognition_motif_domain
SI65_07756	RNA_transport
SI65_07768	Acyltransferase_ChoActase
SI65_07795	Protein_of_unknown_function_DUF3758
SI65_07804	Protein_synthesis_factor,_GTP-binding
SI65_07805	Zinc_finger,_C3HC4_RING-type

SI65_07809	Glycine_cleavage_H-protein
SI65_07816	Chitin_synthase_III_catalytic_subunit
SI65_07817	Glutamine_amidotransferase,_class-II
SI65_07824	Dynamin_central_domain
SI65_07826	Ribosomal_protein_L18e/L15P
SI65_07831	Src_homology-3_domain
SI65_07843	Peptidase_M16
SI65_07882	Surfeit_locus_4
SI65_07884	Metallo-dependent_phosphatase
SI65_07890	GOLD
SI65_07903	Ribonuclease_II/R
SI65_07916	Ion_transport
SI65_07920	D-tyrosyl-tRNA(Tyr)_deacylase
SI65_07938	mRNA_splicing_factor,_thioredoxin-like_U5_snRNP
SI65_07939	Plus-3
SI65_07967	Ubiquinone_biosynthesis_protein_Coq7
SI65_07976	U3_small_nucleolar_RNA-associated_protein_15
SI65_07991	PRELI/MSF1
SI65_07994	Maf1_regulator
SI65_08001	Domain_of_unknown_function_DUF1692
SI65_08022	Ribosome_biogenesis_protein_Nop16
SI65_08029	Zinc_finger,_ZZ-type
SI65_08034	DNA_pol_alpha_N
SI65_08036	Yip1_domain
SI65_08051	DEAD/DEAH-box_helicase
SI65_08055	Retrieval_of_early_ER_protein_Rer1
SI65_08065	Actin-like
SI65_08071	Glycosyltransferase,_ALG10
SI65_08072	Ribosomal_protein_L47,_mitochondrial
SI65_08074	GDP_dissociation_inhibitor
SI65_08078	ATPase,_AAA-type,_core
SI65_08082	RNA_polymerase_II_accessory_factor,_Cdc73
SI65_08087	Glycosyl_transferase,_family_4
SI65_08099	Aconitase_A
SI65_08103	Eukaryotic_type_KH-domain
SI65_08109	Methyltransferase-related
SI65_08114	Domain_of_unknown_function_DUF619
SI65_08117	Nucleolar_GTP-binding_1
SI65_08130	Adenylosuccinate_lyase_
SI65_08132	Biotin-protein_ligase
SI65_08145	Mitochondrial_genome_maintenance_MGM101
SI65_08178	Protein_of_unknown_function_DUF833
SI65_08186	Glutamyl/glutaminyl-tRNA_synthetase,_class_Ic,_catalytic_domain
SI65_08240	Bacterial_Fmu_(Sun)/eukaryotic_nucleolar_NOL1/Nop2p

SI65_08243	tRNA-dihydrouridine_synthase
SI65_08248	Gtr1/RagA_G_protein
SI65_08259	Beta-lactamase-like
SI65_08261	Vacuolar_protein_sorting-associated,_VPS28
SI65_08278	DNA/RNA_helicase,_DEAD/DEAH_box_type
SI65_08280	Pyridine_nucleotide-disulphide_oxidoreductase
SI65_08286	Auxin_efflux_carrier
SI65_08290	HEAT
SI65_08294	Clathrin_adaptor,_mu_subunit
SI65_08299	Fatty_acid_hydroxylase
SI65_08303	CDP-alcohol_phosphatidyltransferase
SI65_08311	Phosphatidylserine_decarboxylase-related
SI65_08334	HEAT // Importin-beta
SI65_08341	Maf-like_protein
SI65_08344	CDC45_family
SI65_08353	WD40_repeat,_subgroup
SI65_08365	GTP1/OBG_subdomain
SI65_08387	Poly(A)_polymerase,_central_domain
SI65_08419	Prefoldin_beta-like
SI65_08449	K_Homology,_type_1,_subgroup
SI65_08456	ATPase,_F1/V1/A1_complex,_alpha/beta_subunit
SI65_08466	Serine/threonine-protein_kinase-like_domain
SI65_08469	GrpE_nucleotide_exchange_factor
SI65_08471	Arginine-tRNA-protein_transferase
SI65_08498	NUDIX_hydrolase_domain
SI65_08499	BING4,_C-terminal
SI65_08500	Ribosome_biogenesis_protein_BMS1
SI65_08512	protein_transport
SI65_08517	Pyridine_nucleotide-disulphide_oxidoreductase,__
SI65_08522	Translocation_protein_Sec62
SI65_08527	Acyltransferase_ChoActase
SI65_08530	Aminotransferase_class-III
SI65_08533	Diphthamide_synthesis,_DPH1/DHP2
SI65_08536	ATPase,_F1_complex,_OSCP/delta_subunit
SI65_08544	Neugrin-related
SI65_08546	ATPase,_AAA-type,_core
SI65_08547	Ribosomal_protein_L10e/L16
SI65_08552	BRCT
SI65_08589	Legume-like_lectin
SI65_08613	Histone_acetyl_transferase_HAT1_N-terminal
SI65_08650	Cullin,_N-terminal
SI65_08652	Armadillo-type_fold
SI65_08657	PBS_lyase_HEAT-like_repeat
SI65_08658	Protein_of_unknown_function_DUF3351

SI65_08664	P60-like
SI65_08673	Protein_of_unknown_function_DUF652
SI65_08686	Aminoacyl-tRNA_synthetase,_class_II_(D/K/N)
SI65_08689	Ribophorin_I
SI65_08708	Riboflavin_kinase_domain,_bacterial/eukaryotic
SI65_08718	COG4_transport
SI65_08719	WD40_repeat,_subgroup
SI65_08728	Protein_of_unknown_function_DUF3661,_vaculolar_transmembrane
SI65_08739	Bacterial_NAD-glutamate_dehydrogenase
SI65_08740	PRP1_splicing_factor,_N-terminal
SI65_08741	Ribosomal_RNA_adenine_methylase_transferase
SI65_08745	Rab-GAP/TBC_domain
SI65_08751	Actin-like
SI65_08752	Mediator_complex,_subunit_Med14
SI65_08756	DNA_polymerase_alpha/epsilon,_subunit_B
SI65_08757	DWNN_domain
SI65_08764	Histidine_biosynthesis
SI65_08765	Replication_factor-A_protein_1
SI65_08767	Peptidase_C19,_ubiquitin_carboxyl-terminal_hydrolase_2
SI65_08769	Adenosine/AMP_deaminase
SI65_08772	Src_homology-3_domain
SI65_08777	Rad17
SI65_08784	Translation_initiation_factor_SUI1
SI65_08864	Exocyst_complex_component_Sec10
SI65_08890	Flavodoxin/nitric_oxide_synthase
SI65_08891	CCCH_zinc_finger
SI65_08892	CS_domain
SI65_08902	Snf7
SI65_08904	Peptidase_C19,_ubiquitin_carboxyl-terminal_hydrolase_2
SI65_08914	Vps16
SI65_08916	Protein_synthesis_factor,_GTP-binding
SI65_08918	Pre-mRNA-splicing_factor_19
SI65_08920	Ribosomal_protein
SI65_08921	Ribosomal_protein_L10/acidic_P0
SI65_08922	Protein_of_unknown_function_DUF3722
SI65_08926	Triosephosphate_isomerase
SI65_08943	Dynamin,_GTPase_domain
SI65_08948	Protein_phosphatase_2C-like
SI65_08951	Inositol_polyphosphate_kinase
SI65_08977	Glycosyl_transferase,_family_1
SI65_08979	Deoxyhypusine_synthase
SI65_08982	Bacterial_Fmu_(Sun)
SI65_08994	Zinc_finger,_XPA-type,_conserved_site
SI65_08995	Eukaryotic_initiation_factor_3,_gamma_subunit

SI65_09065	ARP2/3_complex,_p21-Arc_subunit
SI65_09070	Zinc_finger,_C3HC4_RING-type // Zinc_finger,_CCCH-type
SI65_09078	DNA-binding_SAP
SI65_09094	Domain_of_unknown_function_DUF625
SI65_09102	Ankyrin_repeat-containing_domain
SI65_09103	Helix-loop-helix_DNA-binding_domain
SI65_09112	Sec8_exocyst_complex_component_specific_domain
SI65_09113	Origin_recognition_complex,_subunit_2
SI65_09114	Arginine_biosynthesis_protein_ArgJ
SI65_09121	SecY_protein // Translocon_Sec61/SecY,_plug_domain
SI65_09137	Glycosyl_transferase,_family_2
SI65_09142	Initiator_tRNA_phosphoribosyl_transferase
SI65_09192	PLAA_family_ubiquitin_binding,_PFU // WD40_repeat,_subgroup // PUL
SI65_09193	WD40_repeat,_subgroup // Small-subunit_processome,_Utp12
SI65_09217	Arp2/3_complex,_34kDa_subunit_p34-Arc
SI65_09224	RNA_polymerase_I_specific_transcription_initiation_factor_RRN3
SI65_09233	Ubiquitin-associated/translation_elongation_factor_EF1B,_N-terminal
SI65_09260	Ubiquitin_fusion_degradation_protein_UFD1
SI65_09279	Brix_domain
SI65_09284	Cytochrome_c1 // ATPase,_AAA-type,_core
SI65_09285	Exocyst_complex_component_Sec10 // CAP_domain
SI65_09291	Translation_initiation_factor_2A,_beta_propellor-like_domain // RNA_recognition_motif_domain
SI65_09307	Mitochondrial_import_protein_Pam17
SI65_09323	Serine/threonine-protein_kinase-like_domain
SI65_09349	Phox_homologous_domain
SI65_09356	ATPase,_AAA-type,_core
SI65_09398	Lyase_1,_N-terminal
SI65_09461	cellular_response_to_stress
SI65_09463	Methionine_synthase,_vitamin-B12_independent
SI65_09507	WD40_repeat,_subgroup
SI65_09515	Membrane_protein,Tapt1/CMV_receptor
SI65_09522	Domain_of_unknown_function_DUF21
SI65_09539	Exonuclease,_RNase_T/DNA_polymerase_III
SI65_09546	K_Homology,_type_1,_subgroup
SI65_09549	Zinc_finger,_FYVE-type
SI65_09553	WD40_repeat,_subgroup
SI65_09555	Pre-rRNA-processing_protein_TSR2
SI65_09566	Pr_beta_C // Proteasome,_subunit_alpha/beta
SI65_09571	DNA/RNA_helicase,_DEAD/DEAH_box_type
SI65_09576	Dopey,_N-terminal
SI65_09592	S-adenosyl-L-methionine-dependent_methyltransferases
SI65_09611	Zinc_finger,_FYVE-type
SI65_09624	Chs5p-Arf1p_binding

SI65_09636	Peptidase_S24/S26A/S26B
SI65_09677	Phosphatidate_cytidylyltransferase
SI65_09687	ATPase,_V0/A0_complex,_subunit_C/D
SI65_09691	Pseudouridine_synthase_I,_TruA,_alpha/beta_domain
SI65_09698	Sulphate_transporter
SI65_09699	Translation_initiation_factor_2,_alpha_subunit
SI65_09729	Transcription_factor_TFIIB,_cyclin-related
SI65_09762	Acetohydroxy_acid_isomeroreductase
SI65_09778	DNA-binding_HORMA
SI65_09779	Protein_synthesis_factor,_GTP-binding
SI65_09784	Aminotransferase,_class_V/Cysteine_desulfurase
SI65_09785	Src_homology-3_domain
SI65_09829	Putative_transmembrane_protein
SI65_09851	Helicase
SI65_09858	Glucose-6-phosphate_dehydrogenase
SI65_09871	Translation_initiation_factor_IF2/IF5
SI65_09876	Multiprotein_bridging_factor_1
SI65_09877	WD40_repeat,_subgroup
SI65_10029	Nucleoporin,_Nup133/Nup155-like
SI65_10034	Exportin-1/Importin-beta-like
SI65_10051	Vacuolar_sorting_protein_39
SI65_10063	2-oxoacid_dehydrogenase_acyltransferase
SI65_10158	Ribosomal_protein_S8e
SI65_10160	Serine/threonine-protein_kinase-like_domain
SI65_10164	Fumarylacetoacetase,_C-terminal-like
SI65_10174	Protein_of_unknown_function_DUF2838
SI65_10211	ATPase,_V1
SI65_10321	E3_binding

Table S3 Homology Genes of Mycotoxin Biosynthesis in *Aspergillus cristatus* Genome.

Gene name	Function	Gene ID	E-value of BLASTp	Score-value of InParanoid	Low osmolarity expression (FPKM)	High osmolarity expression (FPKM)	Scaffold position	Reference species	Reference sequence	Reference paper
1. Aflatoxin biosynthesis gene cluster										
aflA	fatty acid synthase beta subunit	SI65_07514	0	1281	156.131	92.693	9	*A. parasitic* ATCC 56775	AY371490	15
aflB	fatty acid synthase alpha subunit	SI65_07515	0	1378	192.71	81.4521	9	*A. parasitic* ATCC 56775	AY371490	15
aflC	polyketide synthase	SI65_03945	0	1469	1.63946	3.58785	3	*A. parasitic* ATCC 56775	AY371490	15
aflE	reductase/dehydrogenase	SI65_01324	3.00E-156	436	0.210877	0	1	*A. parasitic* ATCC 56775	AY371490	15
aflF	conversion of norsolorinic acid to averantin	SI65_08200	0	508	4.44143	0.0736028	12	*A. parasitic* ATCC 56775	AY371490	15
aflG	cytochrome P450 monooxygenase	SI65_04769	9.00E-113	339	22.7455	5.61599	4	*A. parasitic* ATCC 56775	AY371490	15
aflH	short chain alcohol	SI65_01320	2.00E-33	123	1.09516	3.60865	1	*A. parasitic* ATCC 56775	AY371490	15

aflI	dehydrogenase conversion of averufin to versiconal hemiacetal acetate	SI65_01458	4.00E-12	114	41.0722	433.568	1	*A. parasitic* ATCC 56775	AY371490	15
aflJ	esterase	SI65_00063	8.00E-26	105	11.7533	11.4015	1	*A. parasitic* ATCC 56775	AY371490	15
aflK	versicolorin B synthase	SI65_07728	1.00E-169	495	0.521224	0.76745	10	*A. parasitic* ATCC 56775	AY371490	15
aflL	desaturase; versicolorin B synthase	SI65_00736	4.00E-104	339	11.9956	0.16493	1	*A. parasitic* ATCC 56775	AY371490	15
aflM	dehydrogenase	SI65_05588	7.00E-73	242	7.21821	1.34713	5	*A. parasitic* ATCC 56775	AY371490	15
aflN	monooxygenase	SI65_08927	2.00E-70	230	0.150057	0	15	*A. parasitic* ATCC 56775	AY371490	15
aflO	O-methyltransferase B	SI65_09917	2.00E-65	210	0.638686	974.212	22	*A. parasitic* ATCC 56775	AY371490	15
aflP	O-methyltransferase A	SI65_09917	6.00E-31	210	0.638686	974.212	22	*A. parasitic* ATCC 56775	AY371490	15
aflQ	oxidoreductase A	SI65_07268	4.00E-56	200	3.25863	40.2202	8	*A. parasitic* ATCC 56775	AY371490	15
aflR	regulatory protein	SI65_02178	7.00E-11	No	47.9058	0.495291	2	*A. parasitic* ATCC 56775	AY371490	15
aflS	transcription	SI65_08570	3.00E-08	No	1569.32	8.5265	13	*A. parasitic*	AY371490	15

	enhancer							ATCC 56775		
aflT	putative ABC transporter	SI65_10225	2.00E-166	498	2.69258	1.19798	27	*A. parasitic* ATCC 56775	AY371490	15
aflU	cytochrome P450 monooxygenase	SI65_10325	3.00E-129	381	6.40419	21.0413	31	*A. parasitic* ATCC 56775	AY371490	15
aflV	cytochrome P451 monooxygenase	SI65_05426	1.00E-80	275	342.311	51.1166	5	*A. parasitic* ATCC 56775	AY371490	15
aflW	cytochrome P450 monooxygenase	SI65_05998	5.00E-109	334	0.135864	4.55137	6	*A. parasitic* ATCC 56775	AY371490	15
aflX	oxidoreductase B	SI65_01458	6.00E-31	114	41.0722	433.568	1	*A. parasitic* ATCC 56775	AY371490	15
aflY	hypothetical protein	SI65_03953	3.00E-98	298	129.926	61.8284	3	*A. parasitic* ATCC 56775	AY371490	15
**2. Sterigmatocystin biosynthesis Gene Cluster**										
aflR	regulatory protein	SI65_02178	3.00E-08	No	47.9058	0.495291	2	*A. nidulans* FGSC 26	U34740.1	20
stcA	putative polyketide synthase	SI65_03945	0	1469	1.63946	3.58785	3	*A. nidulans* FGSC 26	U34740.1	20
stcB	putative polyketide synthase	SI65_05771	9.00E-81	275	32.432	0.116221	5	*A. nidulans* FGSC 26	U34740.1	20
stcC	putative	SI65_00041	6.00E-34	125	1.53917	68.4604	1	*A. nidulans*	U34740.1	20

	peroxidase							FGSC 26		
stcE	putative ketoreductase	SI65_07450	4.00E-42	181	11.8378	0.240843	9	*A. nidulans* FGSC 26	U34740.1	20
stcF	putative p450 monooxygenase	SI65_00736	9.00E-108	339	11.9956	0.16493	1	*A. nidulans* FGSC 26	U34740.1	20
stcI	putative lipase/esterase	SI65_00063	9.00E-21	105	11.7533	11.4015	1	*A. nidulans* FGSC 26	U34740.1	20
stcJ	putative fatty acid synthase alpha	SI65_07514	1.00E-79	1281	156.131	92.693	9	*A. nidulans* FGSC 26	U34740.1	20
stcL	putative p450 monooxygenase	SI65_02168	9.00E-109	352	0.832441	0.229922	2	*A. nidulans* FGSC 26	U34740.1	20
stcO	similar to A. parasiticus putative aflatoxin biosynthesis protein	SI65_01458	3.00E-13	No	41.0722	433.568	1	*A. nidulans* FGSC 26	U34740.1	20
stcQ	similar to *A. parasiticus* putative aflatoxin biosynthesis protein	SI65_01458	1.00E-30	114	41.0722	433.568	1	*A. nidulans* FGSC 26	U34740.1	20
stcS	putative p450 monooxygenase	SI65_08927	3.00E-61	230	0.150057	0	15	*A. nidulans* FGSC 26	U34740.1	20
stcT	putative translation elongation factor 1	SI65_03452	4.00E-50	173	363.157	93.8825	3	*A. nidulans* FGSC 26	U34740.1	20

stcU	gamma putative ketoreductase	SI65_05588	2.00E-70	242	7.21821	1.34713	5	*A. nidulans* FGSC 26	U34740.1	20
stcV	putative dehydrogenase	SI65_01324	2.00E-148	436	0.210877	0	1	*A. nidulans* FGSC 26	U34740.1	20
stcW	putative FAD-containing monooxygenase	SI65_05998	1.00E-95	334	0.135864	4.55137	6	*A. nidulans* FGSC 26	U34740.1	20
stcK	putative fatty acid synthase beta subunit	SI65_07515	0	1378	192.71	81.4521	9	*A. nidulans* FGSC 26	U34740.1	20
**3. Gliotoxin biosynthesis Gene Cluster**										
gliA	MFS gliotoxin efflux transporter	SI65_10225	2.00E-141	No	2.69258	1.19798	27	*A. fumigates* Af293	AY838877	17
gliK	Gamma-glutamyl cyclotransferase	SI65_09278	4.00E-27	110	97.7115	74.1883	17	*A. fumigates* Af293	AY838877	17
gliG	glutathione S-transferase	SI65_05852	3.00E-23	94	27.541	80.4417	5	*A. fumigates* Af293	AY838877	17
gliM	O-methyltransfera se	SI65_08573	7.00E-18	No	6.21553	0.245922	13	*A. fumigates* Af293	AY838877	17
gliP	nonribosomal peptide synthase	SI65_10013	0	924	369.781	0.957772	24	*A. fumigates* Af293	AY838877	17
gliJ	membrane dipeptidase	SI65_10086	1.00E-88	271	123.203	32.2374	25	*A. fumigates* Af293	AY838877	17
gliI	Aminotransferase	SI65_00213	2.00E-50	173	70.5362	139.984	1	*A. fumigates* Af293	AY838877	17

gliZ	GAL4-like Zn2Cys6 binuclear cluster DNA-binding domain	SI65_02833	3.00E-08	No	2.10206	0.978578	2	*A. fumigates* Af293	AY838877	17
gliC	cytochrome P450 oxidoreductase	SI65_02538	3.00E-17	No	47.8642	7.62516	2	*A. fumigates* Af293	AY838877	17
gliT	thioredoxin reductase	SI65_03923	3.00E-41	146	100.492	2.30086	3	*A. fumigates* Af293	AY838877	17
gliF	cytochrome P450 oxidoreductase	SI65_03683	3.00E-53	184	4.98223	1.12875	3	*A. fumigates* Af293	AY838877	17
gliN	methyltransferase	SI65_09564	3.00E-17	82	5.04975	0.359262	4	*A. fumigates* Af293	AY838877	17
**4. Zearalenone biosynthesis Gene Cluster**										
ACA	Ca2+ ATPase	SI65_02821	0	1181	6.51965	7.5613	2	*F. graminearum PH-1*	DQ019316	19
ALD	aldehyde dehydrogenase	SI65_10177	1.00E-127	378	29.6874	9.28873	26	*F. graminearum PH-1*	DQ019316	19
HET	heterokaryon incompatibility protein	SI65_03339	2.00E-24	No	22.4354	4.11164	3	*F. graminearum PH-1*	DQ019316	19
KAT	K+ channel protein	SI65_08176	8.00E-127	362	1.47241	0.478249	12	*F. graminearum PH-1*	DQ019316	19
MCT	monocarboxylate	SI65_03765	5.00E-79	273	1.51132	0.659147	3	*F. graminearum*	DQ019316	19

	transporter like-protein							*PH-1*		
NPS	non-ribosomal peptide synthetase	SI65_05056	0	937	52.4799	20.7725	4	*F. graminearum PH-1*	DQ019316	19
PKS13	polyketide synthase(required for zearalenone production)	SI65_03276	0	1027	10.3076	0.0669255	3	*F. graminearum PH-1*	DQ019316	19
PKS4	polyketide synthase(required for zearalenone production)	SI65_02847	0	No	1326.82	1.82172	2	*F. graminearum PH-1*	DQ019316	19
STK	protein kinase Eg2-like protein	SI65_03555	2.00E-135	380	24.4849	14.6216	3	*F. graminearum PH-1*	DQ019316	19
ZEB1	isoamyl alcohol oxidase(required for zearalenone production)	SI65_03320	1.00E-180	532	14.7469	25.0216	3	*F. graminearum PH-1*	DQ019316	19
ZEB2	bZIP domain-containing transcription factor(required for zearalenone)	SI65_03015	1.00E-06	No	193.251	160.526	2	*F. graminearum PH-1*	DQ019316	19
**5.Fumonisin biosynthesis Gene Cluster**										
cpm1	cytochrome P450 monooxygenase	SI65_09993	1.00E-103	320	20.663	7.19406	23	*F. oxysporum* FRCO-1890	EU449979	16

fum1	Acyl transferase domain in polyketide synthase(PKS) enzymes	SI65_02164	0	1921	0.290841	0.430763	2	*F. oxysporum* FRCO-1890	EU449979	16
fum10	Acyl-CoA synthetase (AMP-forming)/AMP-acid ligaseII	SI65_03213	8.00E-95	292	44.708	106.816	2	*F. oxysporum* FRCO-1890	EU449979	16
fum11	mitochondrial tricarboxylate transporter	SI65_06193	2.00E-112	321	37.5793	18.6847	6	*F. oxysporum* FRCO-1890	EU449979	16
fum13	fumonisin cluster-short-chain Dehydrogenase(fumonisin 3-keto	SI65_00023	8.00E-29	113	12.1862	163.421	1	*F. oxysporum* FRCO-1890	EU449979	16
fum14	fumonisin cluster-peptidsynthase condensation domain(nrps c domain like)	SI65_01682	9.00E-20	91	0.130755	0.0488016	1	*F. oxysporum* FRCO-1890	EU449979	16
fum15	fumonisin cluster-P450 monooxygenase	SI65_09492	3.00E-135	399	7.0156	1.43238	19	*F. oxysporum* FRCO-1890	EU449979	16

fum16	fumonisin cluster-fatty acyl-CoA Synthase	SI65_06526	0	597	124.534	139.017	7	*F. oxysporum* FRCO-1890	EU449979	16
fum17	hypothetical protein	SI65_04379	6.00E-106	351	59.0469	34.5105	4	*F. oxysporum* FRCO-1890	EU449979	16
fum18	fumonisin cluster-longevity assurance factor	SI65_03168	2.00E-84	272	50.1691	17.5612	2	*F. oxysporum* FRCO-1890	EU449979	16
fum19	fumonisin biosynthetic ABC transporter	SI65_10152	0	787	20.0998	11.9259	26	*F. oxysporum* FRCO-1890	EU449979	16
fum2	fumonisin cluster-P450 monooxygenase	SI65_02168	2.00E-117	352	0.832441	0.229922	2	*F. oxysporum* FRCO-1890	EU449979	16
fum21	fumonisin biosynthetic transcription factor	SI65_05581	7.00E-13	107	48.3784	53.9968	5	*F. oxysporum* FRCO-1890	EU449979	16
fum3	fumonisin cluster-dioxygenas e(C-5 hydroxylase)	SI65_05609	3.00E-16	76	79.584	10.3441	5	*F. oxysporum* FRCO-1890	EU449979	16
fum6	fumonisin cluster-P450 monooxygenase	SI65_07364	0	742	26.6113	33.2308	9	*F. oxysporum* FRCO-1890	EU449979	16
fum7	fumonisin biosynthetic	SI65_01078	9.00E-89	277	1.90784	7.59041	1	*F. oxysporum* FRCO-1890	EU449979	16

	dehydrogenase									
fum8	fumonisin cluster-aminotransferase	SI65_07829	9.00E-39	145	69.1476	55.0069	10	*F. oxysporum* FRCO-1890	EU449979	16
**6. Ochratoxin biosynthesis Gene Cluster**										
NPS	non-ribosomal peptide synthetase	SI65_05056	0	937	52.4799	20.7725	4	*P. nordicum* BFE487	AY557343	18
ASP	alkaline serine protease	SI65_09887	0	1249	159.453	3.50237	22	*P. nordicum* BFE487	AY557343	18
otapksPN	ochratoxin A polyketide synthase	SI65_09913	3.00E-124	369	26.5317	10.802	22	*P. nordicum* BFE487	AY557343	18
