# Supplementary material for: Comparative genomic and transcriptomic analyses of the Fuzhuan brick tea-fermentation fungus Aspergillus cristatus
Source: BMC Genomics. 2016 Jun 7;17:428. doi: 10.1186/s12864-016-2637-y (PMC4895823; doi:10.1186/s12864-016-2637-y)
Supplement: Additional file 2: Figure S1. — Results of MAT1-1-1 and MAT1-2-1 amino acid sequence analysis of members of the genus Aspergillus using Jalview version 2.0 [32]. Figure S2. Detection of six mycotoxins by HPLC. (PDF 401 kb) [file 12864_2016_2637_MOESM2_ESM.pdf]

Additional Files 1

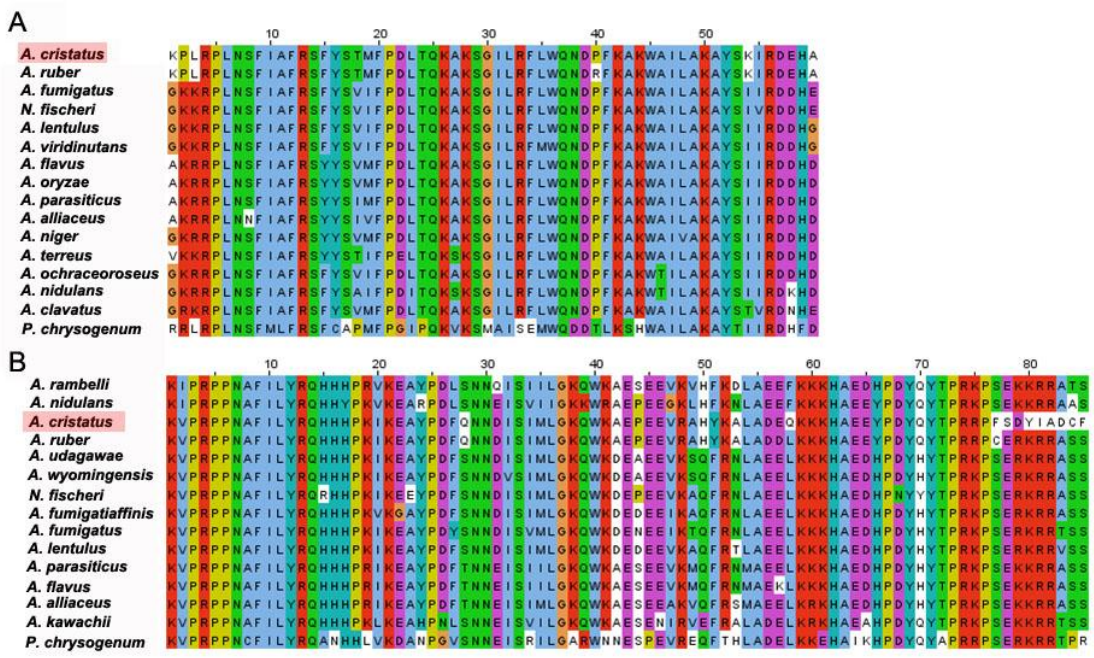

Figure S1 Results of *MAT1-1-1* and *MAT1-2-1* amino acid sequence analysis of members of the genus *Aspergillus* using Jalview version 2.0 [32].

A. *MAT1-1-1* amino acid sequence analysis. B. *MAT1-2-1* amino acid sequence analysis.

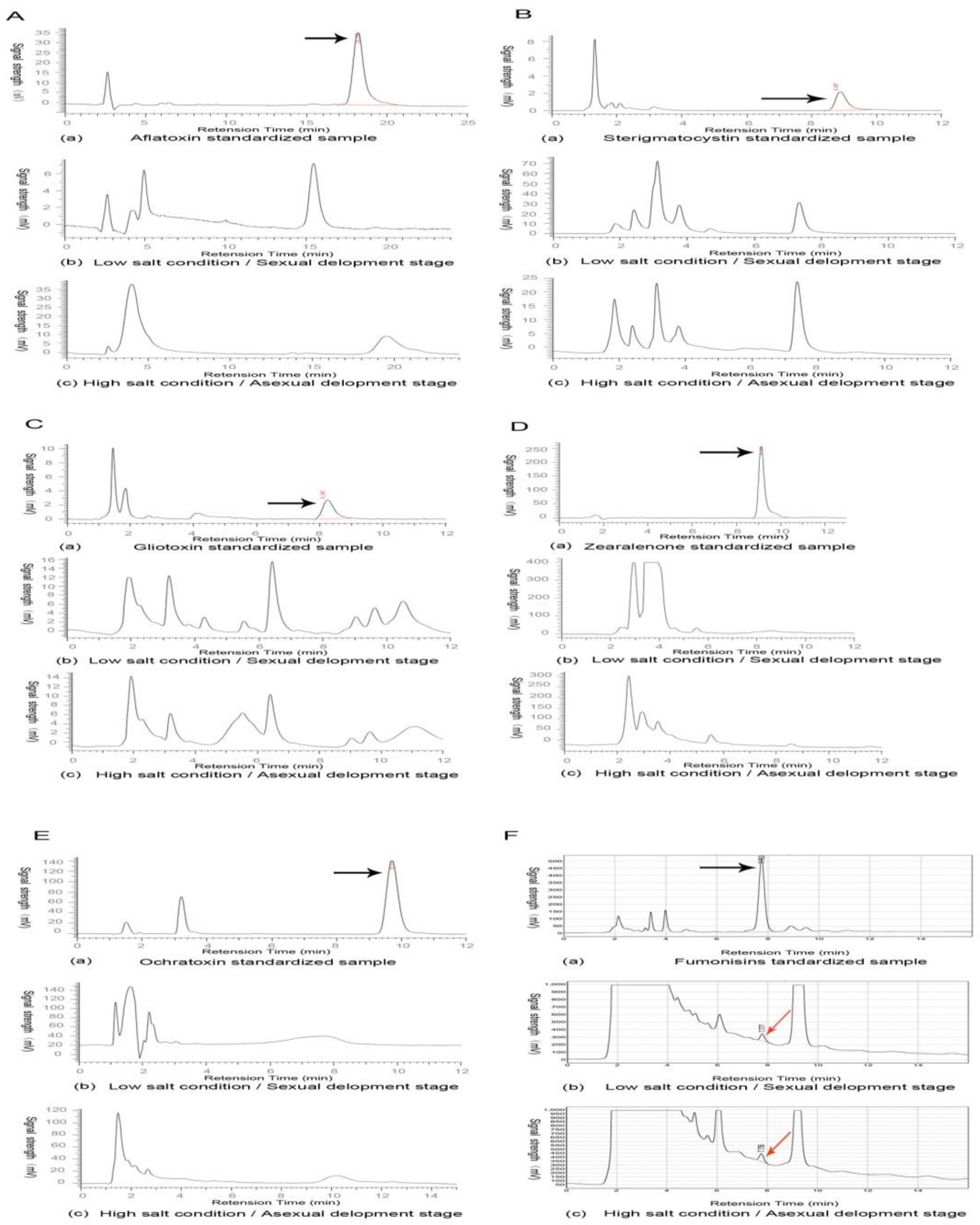

Figure S2 Detection of six mycotoxins by HPLC.

Detection of A. aflatoxin B_1, B. sterigmatocysin, C. gliotoxin, D. zearalenone, E. ochratoxin A, and F. fumonisin B_1. (a) Results of standard samples. (b) Results of detection under low-salt conditions. (c) Results of detection under high-salt conditions. Black and red arrows indicated the signals of the standard products and the samples,

respectively.

Standard solutions were purchased from Pribolab (Pribolab Pte. Ltd, Singapore) and were used in the following concentration ranges: 0.5-10 ng/mL for aflatoxin B_1, 10-500 ng/mL for sterigmatocysin and zearalenone, 50-500 ng/mL for gliotoxin, 2.5-50 ng/mL for ochratoxin A, and 250-5000 ng/mL for fumonisin B_1. These solutions were used to generate standard curves. Sample contents were calculated using the formula $C=(A\times V) / (m\times f)$ [75].
